# Predicting insect outbreaks using machine learning: A mountain pine beetle case study

**DOI:** 10.1002/ece3.7921

**Published:** 2021-09-12

**Authors:** Pouria Ramazi, Mélodie Kunegel‐Lion, Russell Greiner, Mark A. Lewis

**Affiliations:** ^1^ Department of Mathematical and Statistical Sciences University of Alberta Edmonton AB Canada; ^2^ Department of Computing Science University of Alberta Edmonton AB Canada; ^3^ Department of Biological Sciences University of Alberta Edmonton AB Canada; ^4^ Alberta Machine Intelligence Institute Edmonton AB Canada

**Keywords:** future infestations, insect spread, machine learning, mountain pine beetle, predictive ecology, temporal prediction

## Abstract

Planning forest management relies on predicting insect outbreaks such as mountain pine beetle, particularly in the intermediate‐term future, e.g., 5‐year. Machine‐learning algorithms are potential solutions to this challenging problem due to their many successes across a variety of prediction tasks. However, there are many subtle challenges in applying them: identifying the best learning models and the best subset of available covariates (including time lags) and properly evaluating the models to avoid misleading performance‐measures. We systematically address these issues in predicting the chance of a mountain pine beetle outbreak in the Cypress Hills area and seek models with the best performance at predicting future 1‐, 3‐, 5‐ and 7‐year infestations. We train nine machine‐learning models, including two generalized boosted regression trees (GBM) that predict future 1‐ and 3‐year infestations with 92% and 88% AUC, and two novel mixed models that predict future 5‐ and 7‐year infestations with 86% and 84% AUC, respectively. We also consider forming the train and test datasets by splitting the original dataset *randomly* rather than using the appropriate year‐based approach and show that this may obtain models that score high on the test dataset but low in practice, resulting in inaccurate performance evaluations. For example, a *k*‐nearest neighbor model with the actual performance of 68% AUC, scores the misleadingly high 78% on a test dataset obtained from a random split, but the more accurate 66% on a year‐based split. We then investigate how the prediction accuracy varies with respect to the provided history length of the covariates and find that neural network and naive Bayes, predict more accurately as history‐length increases, particularly for future 1‐ and 3‐year predictions, and roughly the same holds with GBM. Our approach is applicable to other invasive species. The resulting predictors can be used in planning forest and pest management and planning sampling locations in field studies.

## Graphical abstract

There are subtle challenges in applying machine‐learning algorithms to forecasting insect outbreaks in the intermediate‐term future. We address these issues and provide step‐by‐step instructions using the case study of the mountain pine beetle outbreak in the Cypress Hills.

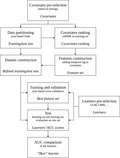

## 1 INTRODUCTION

Forest insect outbreaks can cause huge damage to the environment and economy (Dale et al., 2001; Venier & Holmes, 2010; Walton, 2013). Forest management is, thus, crucial, and includes both prevention and direct control. In Canada, forest management agreement plans are made for five years (Government of Alberta, 2019), and they need an additional year or two for preparation. Therefore, predicting seven years in the future is a reasonable time horizon for planning prevention measures. Making short‐term predictions, e.g., future 1‐year (for a 1‐year life‐cycle insect), via statistical models, such as generalized linear models (GLM) (Oliver et al., 2008; Smolik et al., 2010), is usually straightforward, given the temporal autocorrelation present in ecological systems (Boyce et al., 2010; Otis & White, 1999). Making long‐term predictions, e.g., future 30‐year, is, on the other hand, sometimes feasible via the asymptotic analysis of ecological dynamical systems as they are often attracted to an expected outcome (Ferrari et al., 2014; Hastings et al., 1993; Ramazi et al., 2016; Schaffer & Kot, 1985). However, to the best of our knowledge, except for a few works (e.g., de la Fuente et al., 2018), methods for making accurate intermediate‐term predictions remain mainly untouched, which yields a challenge to ecological modelers. The time scale is too long for the ecological transients to be linked to environmental variability via statistical analyses, yet it is too short for dynamical systems to approach their attractor.

Researchers have, hence, looked to other approaches, especially those in machine learning due to their many successes in a variety of areas. Examples of models include decision trees (Broennimann & Guisan, 2008; Hestir et al., 2008), support vector machines (SVM) (Atkinson et al., 2013), *k*‐nearest neighbors (KNN), Bayesian networks (Bressan et al., 2009), and neural networks (NN) (Worner et al., 2014). However, there are several challenges faced upon predicting future infestations that are rarely addressed in the literature.

First, and foremost, is the identification of proper model evaluation. The typical approach in machine learning is to randomly partition the dataset into a *training* subset, for parameter estimation, and a disjoint *testing*, for performance evaluation. It turns out that this, however, can easily result in sub‐optimal predictors, with misleadingly estimates of accuracy. However, this issue can be solved by choosing an alternative partition of data into training and testing components that better reflects the structure of the task at hand. We now consider a detailed example where we illustrate the issues at hand. Suppose that we would like to predict the presence of infestation at a particular area at year 2024. The available data, is limited to be up to at most the present year, say 2019. So the task is to learn a model that can use data up until year *T*, to predict infestation at year T+5. Correspondingly, the model evaluation must reflect the performance on this particular task – i.e., predicting 5 years in the future. Namely, if the available data for learning the model is from years 2010 to 2019, then the training dataset must include years 2010 to say T=2014 and the test must include only T+5=2019. Thus, there should be a 5‐year gap between the training and testing datasets. If, instead, we were to randomly split the dataset, and both train and test contain observations from the same year, then the evaluation would represent how well the model predicts *current* infestations rather than those in *future*, that is usually a more complex task.

The second challenge is feature (covariate) selection. Given a fixed training set, the addition of more features does not necessarily result in a more accurate predictor. However, by exhaustive searches through possible covariate combinations, such as the exhaustive enumeration of subset (Sokal & Rohlf, 1995) or the step AIC (Venables & Ripley, 2002) we increase the chance of overfitting parameters to the training dataset, and thus, of failing to make accurate predictions on the test dataset.

The third challenge is the history‐length to include for the covariates. Prediction accuracy may improve by using past information (history) regarding the features, e.g., precipitation several years before the year of interest (Preisler et al., 2012). However, is it best to add as much history as possible? The drawback is that adding longer history for each feature also increases exponentially the total number of feature combinations to choose from in model selection, potentially making model selection unwieldy.

We address these three issues with the case study of a mountain pine beetle (MPB) outbreak in the Cypress Hills area in Canada. We have recently investigated the impact of, and relations between, some potential covariates of the MPB infestation using Bayesian networks (Ramazi et al., 2021). Predicting future MPB infestation, however, requires different tools and analysis, which is what we investigate here. In particular, our objectives are to [noitemsep,nolistsep].

1. accurately predict infestation locations at short and intermediate time scales (1, 3, 5, and 7 years in the future) using the machine‐learning models generalized boosted classification tree (GBM), GLM, SVM, Bayesian networks including Naive Bayes (NB) and those obtained by structure learning, KNN, NN, and a mixed model in the form of a GLM of the aforementioned models,
2. systematically choose from the available covariates,
3. examine whether providing more history regarding covariates actually improves future predictions,
4. examine whether the “actual performance” of a model is better estimated by a test dataset obtained from an appropriate year‐based split of the original dataset rather than a test dataset obtained from a random split of the original dataset.

We distinguish our work from studies predicting the geographical extent of species invasions (Broennimann & Guisan, 2008) in large scales, as we focus on a small area, with finer ranges of covariates as in (Aukema et al., 2008; Preisler et al., 2012; Sambaraju et al., 2012).

### 1.1 Mountain pine beetle biology

The mountain pine beetle is an eruptive bark beetle that infests pine forests in western North America. Beetles usually attack susceptible pines within a few hundred meters of their emergence site (Carroll & Safranyik, 2004). However, in rare occasions, they have been reported to engage in a long‐distance dispersal behaviour by getting caught in the wind above the tree canopy and dispersing passively hundreds or thousands of kilometers (Chen & Jackson, 2017; Safranyik & Carroll, 2006). Trees use a defense mechanism consisting of toxic resin exuding from the galleries dug by the beetles (Erbilgin et al., 2017; Raffa & Berryman, 1983). Therefore, a water‐deficit during the tree growing season decrease its defenses abilities against mountain pine beetle (Lusebrink et al., 2016). Summer and winter temperatures affect larvae development and survival in the tree as well as adult emergence and dispersal (Safranyik & Carroll, 2006). The orientation of the slope – i.e., the aspect – would have a similar effect by creating different micro‐climates, thereby affecting beetle development and survival. Lastly, by controlling infestations, managers modify dispersal and survival rates. Thus, the proximity of managed infestations will likely modify the probability of infestation at a certain location.

## 2 MATERIALS AND METHODS

### 2.1 Raw data

We use mountain pine beetle infestation data from the Cypress Hills interprovincial park collected by the Saskatchewan Forest Service between 2006 and 2018 in association with topography, weather, and vegetation variables (Table 1). The variables and data collection and processing are described in details in (Kunegel‐Lion et al., 2020a) and the dataset is available from Dryad at https://doi.org/10.5061/dryad.70rxwdbt9 (Kunegel‐Lion et al., 2020b).

**TABLE 1:** Description of the covariates

Symbol	Description	Unit
$N_{g}$	Northerness defined as the cos of the angle of the average compass direction that the slopes at pixel g face	
$E_{g}$	Easterness defined as the sin of the angle of the average compass direction that the slopes at pixel g face	
$B_{g}$	Distance from the centre of pixel g to the border of the whole area of interest that was initially infested (the dotted red line in Figure S1)	km
$D_{g,t}$	Degree days (sum of daily temperatures above 5.5°C) from fall of year t‐1 to summer of year t	
$T_{g,t}^{\text{min}}$	Lowest minimum daily temperature in winter of year t	°C
$T_{g,t}^{\text{max}}$	Highest maximum daily temperature in July and August of year t	°C
$W_{g,t}$	Average daily wind speed in July and August of year t	km/hr
$R_{g,t}$	Average daily relative humidity in spring of year t	%
$C_{g,t}$	Cold tolerance defined as an index in $\left[0,1\right]$ representing the ability of the larvae to survive the cold season of year t, as defined in (Régnière & Bentz, 2007)	
$I_{g,t}^{\text{Managed}}$	Managed last year infestation defined to be 1 if pixel g includes at least one tree that was infested and managed (controlled) at year t‐1, and 0 otherwise (Figure S2)	
$I_{g,t}^{\text{Missed}}$	Missed last year infestation defined to be 1 if pixel g includes at least one tree that was infested and missed (unmanaged and not controlled) at year t‐1, and 0 otherwise (Figure S2)	
$I_{N_{g},t}^{\text{Missed}}$	Missed neighbors’ last year infestation represents the mountain pine beetles’ ability to disperse at short distances within a stand, defined as $I_{N_{g},t}^{\text{Missed}}=\sum_{i=1}^{3}\frac{1}{2^{i}}\sum_{g^{\prime }\in N_{g}^{i}}I_{g^{\prime },t}^{\text{Missed}}$ $I_{N_{g},t}^{\text{Missed}}\in \left[0,6\right]$ where $N_{g}^{i}$ are those pixels that are essentially at a distance of i×100m from g (Figure S3); for those pixels on or close to the boundary of the park, $N_{g}^{i}$ includes only neighbors within the park	
$I_{N_{g},t}^{\text{Managed}}$	Managed neighbors’ last year infestation defined similarly to $I_{N_{g},t}^{\text{Missed}}$, with the difference that $I_{g^{\prime },t}^{\text{Missed}}$ is replaced by $I_{g^{\prime },t}^{\text{Managed}}$	
$O_{t}$	Phase of the mountain pine beetle outbreak at year t‐1, defined to be 1 (*increase*), 2 (*peak*), or 3 (*decline*)	

### 2.2 Analysis overview

**FIGURE 1:**
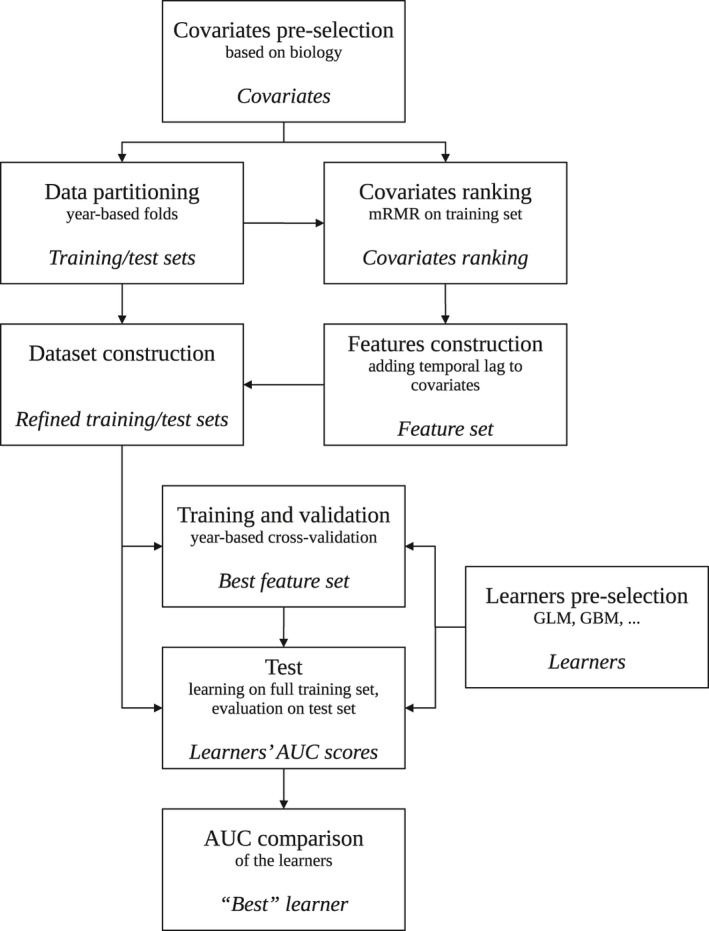
Flowchart representing the method steps. Each square represents a step. Text in italic is the output of the step and used in the following steps

We approach the problem by taking the following steps (Figure 1). First, we define the target variable and choose the covariates based on the biology of the problem. Next, we perform a year‐based partitioning of the dataset to obtain the training and validation datasets. Then we rank the covariates using the mRMR method on the training dataset. We construct feature sets based on the ranked covariates and their historical values and refine the datasets accordingly. Next, we train several learners, including the generalized linear model, on the training dataset and perform year‐based cross‐validation to find the feature set that performs best during the cross‐validation. Finally, we re‐train the learners with their best feature sets on the whole training dataset and compare their performances on the test dataset to obtain the best learner. In what follows, we explain these steps in detail.

### 2.3 Target variable, covariates, and features

We divide the Cypress Hills park area (Figure S1) into a total of *N* = 238,121 squares, each of size 100m×100m, referred to as *pixels*, and label them by integers 1,2,…. Let $I_{g,t}\in \left\{0,1\right\}$ denote the presence of infestation at a pixel g at fall of year t, which is defined to be 1 if there is an infested tree and 0 otherwise. Given a pixel g and year t, the target variable is the presence of infestation at pixel g, r years in the future, i.e., $I_{g,t+r}$, for r=1,3,5 and 7. We consider the following covariate set, consisting of 14 covariates defined in Table 1:

(1) $$X_{g,t}=\left\{N_{g},E_{g},B_{g},D_{g,t},T_{g,t}^{\text{min}},T_{g,t}^{\text{max}},W_{g,t},R_{g,t},C_{g,t},O_{t},I_{N_{g},t}^{\text{Missed}},I_{N_{g},t}^{\text{Managed}},I_{g,t}^{\text{Missed}},I_{g,t}^{\text{Managed}}\right\}.$$

All covariates except for minimum temperature and outbreak phase are taken from (Ramazi et al., 2021). Each covariate is associated with a pixel g and/or a time t. All covariates in $X_{g,t}$ are measured during fall of year t‐1 to summer of year t, except for $I_{g,t}^{\text{Missed}}$, which is determined only after the survey in fall of year t. We, therefore, refer to the covariates in $X_{g,t}$ as those *measured at year* t.

We are interested in predicting infestations r years into the future based on h years of data. Thus, the prediction for $I_{g,t+r}$, uses the covariates measured at years t,t‐1,…,t‐h+1, i.e., $X_{g,t}$, $X_{g,t‐1},\ldots ,X_{g,t‐h+1}$, for $h\in \left\{1,\ldots ,5\right\}$. That is, using data of a specific pixel, say pixel 17, from 2010 to 2012, predict whether that pixel will be infested at 2015 – i.e., given $X_{17,2010},X_{17,2011},X_{17,2012}$, predict $I_{17,2015}$ (so g=17, t=2012, r=3, and h=3). We define the set of *features* as $F_{g,t}^{h}:=X_{g,t}\cup X_{g,t‐1}\cup \ldots \cup X_{g,t‐h+1}$. Note that we are distinguishing ‘covariates’ from ‘features’: covariates are only those in $X_{g,t}$, but both the covariates and their historical values are referred to as features. ‘The best’ predictive model may only use a subset of these features, as discussed in the following sections. The variable h determines the total number of years used for prediction, which we refer to as the *history‐length* and have limited it to be no more than 5 years. Clearly, historical values of the non‐temporal covariates – i.e., $N_{g},E_{g}$ and $B_{g}$ (Table 1)– are the same as their current values.

### 2.4 Partitioning the data into train and test

**FIGURE 2:**
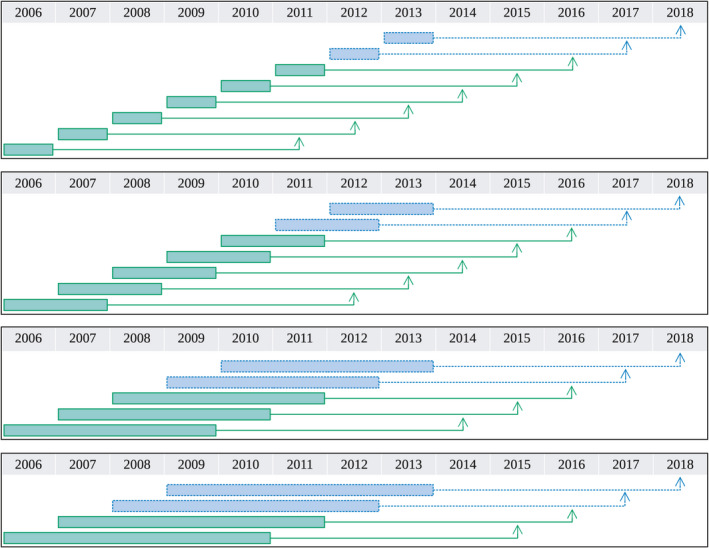
Dataset partition for r=5 years in the future. The boxes indicate which years the covariates are measured (t‐h+1,…,t), and the arrows point to the year at which we predict infestation (t+r). So the length of each box represents h and the length from the box to the arrow represents r. Green solid lines represent the training dataset whereas blue dashed lines represent the testing dataset. From top to bottom: 1‐, 2‐, 4‐, and 5‐year history‐length

Having the goal of estimating infestations in future years, we set the testing dataset $D_{\text{test}}$ to be the data with the target variable from the last two available years – i.e., $\left(t+r\right)\in \left\{2017,2018\right\}$ – and let the training dataset $D_{\text{train}}$ to be the data with the target variable from the remaining years – i.e., $\left(t+r\right)\in \left\{2005+h+r,\ldots ,2015,2016\right\}$; *n.b*., they are yearly disjoint. The datasets are clearly different for each history‐length h (Figure 2). Correspondingly, given each history‐length h and future‐prediction‐length r, we will have the train and test datasets $D_{\text{train}}^{r,h}$ and $D_{\text{test}}^{r,h}$. In both the training and testing datasets, the covariates for each instance at year t are measured up to h‐1 years before, i.e., t‐h+1,t‐h+2,…,t, and the target variable is measured at year t+r. Hence, the training dataset is formed by the union of ‘blocks of instances’ at years t=2006+h‐1,…,2016‐r, and the testing dataset is formed by those at years t=2017‐r and 2018‐r.

### 2.5 Feature selection

To find that set of features resulting in the highest prediction accuracy over the underlying distribution, one may exhaustively search through all possible combinations of the features in the training dataset. Namely, to predict $I_{g,t+r}$, we can choose from the 14×h features in $F_{t}^{h}$: 14 covariates in $X_{g,t}$, each with a history‐length of h years. For h=5, this results in a total of $2^{14\times 5}=1e21$ combinations of features, which is not only infeasible to search through, but also quite likely to result in overfitting the training dataset.

We limit our search over the features as follows. First, given the target variable $I_{g,t+r}$, we rank the covariates in $X_{g,t}$ based on all pixels g and all years t in $D_{\text{train}}^{r,h}$, using the *minimum redundancy maximum relevance (mRMR)* method (Ding & Peng, 2005), which prioritizes covariates that have a strong correlation to the target variable (maximum relevance), but are mutually far from each other (minimum redundancy). We use the package mRMRe in R (De Jay et al., 2013). This results in an ordering $X_{t}^{1}≻X_{t}^{2}≻\ldots ≻X_{t}^{14}$ of the covariates, where $X_{t}^{i}$‘s are the elements of $X_{g,t}$ in (1) (the notation g is omitted from $X_{t}^{i}$ for simplicity), and A≻B implies that A is ranked over B in the mRMR ranking (see Eq. S1 for an example). The ranking can be different for each future‐number‐of‐years r.

Second, we consider the following 14 covariate sets:

$$\underset{X_{g,t}^{1}}{\underbrace{\left\{X_{t}^{1}\right\}}},\underset{X_{g,t}^{2}}{\underbrace{\left\{X_{t}^{1},X_{t}^{2}\right\}}},\underset{X_{g,t}^{3}}{\underbrace{\left\{X_{t}^{1},X_{t}^{2},X_{t}^{3}\right\}}}\ldots ,\underset{X_{g,t}^{14}=X_{g,t}}{\underbrace{\left\{X_{t}^{1},X_{t}^{2},\ldots ,X_{t}^{14}\right\}}}.$$

Third, for each of the above 14 combinations, we provide up to 5 years of history‐length. Therefore, given a number‐of‐covariates $c\in \left\{1,\ldots ,14\right\}$ and history‐length $h\in \left\{1,\ldots ,5\right\}$, we obtain a feature set $F_{g,t}^{r,h,c}:=X_{g,t}^{c}\cup \ldots \cup X_{g,t‐h+1}^{c}$, containing a total of c×h features (Table 2). Overall, for each feature r years, we will be training our predictive models on a total of 14×5=70 combinations of features. Note this is significantly smaller than the complete set of $2^{14\times 5}$ possible subsets.

**TABLE 2:** The covariate set $F_{g,t}^{r,h,c}$ for history‐length h and number‐of‐features c

f	1‐year history	2‐year history	…	5‐year history
1	$\left\{X_{t}^{1}\right\}$	$\left\{X_{t}^{1},X_{t‐1}^{1}\right\}$	…	$\left\{X_{t}^{1},\ldots ,X_{t‐4}^{1}\right\}$
2	$\left\{X_{t}^{1},X_{t}^{2}\right\}$	$\left\{X_{t}^{1},X_{t}^{2},X_{t‐1}^{1},X_{t‐1}^{2}\right\}$	…	$\left\{X_{t}^{1},X_{t}^{2},\ldots ,X_{t‐4}^{1},X_{t‐4}^{2}\right\}$
⁝	⁝	⁝	⋱	⁝
14	$\left\{X_{t}^{1},\ldots ,X_{t}^{14}\right\}$	$\left\{X_{t}^{1},\ldots ,X_{t}^{14},\ldots ,X_{t‐1}^{1},\ldots ,X_{t‐1}^{14}\right\}$	…	$\left\{X_{t}^{1},\ldots ,X_{t}^{14},\ldots ,X_{t‐4}^{1},\ldots ,X_{t‐4}^{14}\right\}$

Fourth, we construct a dataset specific to each of the feature sets as follows. The dataset corresponding to feature‐set $F_{g,t}^{r,h,c}$, denoted by $D^{r,h,c}$, consists of c×h columns – one for each feature – plus one column for the target variable $I_{g,t+r}$, over all pixels g=1,…,N, and all years t=2006+h‐1,2006+h,2006+h+1,…,2018‐r, resulting in a total of $N\times \left(14‐r‐h\right)$ rows (Figure 2). The train and test datasets $D_{\text{train}}^{r,h,c}$ and $D_{\text{test}}^{r,h,c}$ are obtained correspondingly from $D_{\text{train}}^{r,h}$ and $D_{\text{test}}^{r,h}$.

### 2.6 Learning algorithms

We use the following learners to obtain the predictive models (Table 3): SVM, GLM, GBM, NB, Chow‐Liu (CL) algorithm for finding a Bayesian network, incremental association Markov blanket (IAMB) algorithm for finding a Bayesian network, KNN, NN, and a mixed model (MM) in the form of a logistic regression of the infestation probabilities provided by each of the 8 previous models.

**TABLE 3:** Description of the algorithms

Name	Description	R Package information
Support vector machine (SVM)	Constructs a hyper‐plane in the covariate space to classify the target variable (Cortes & Vapnik, 1995). A linear SVM classifies the presence of MPB as $P\left(I_{g,t+r}\right)=1$ if $\theta \cdot X+\theta^{0}\geq 0$ and $P\left(I_{g,t+r}\right)=0$ if $\theta \cdot X+\theta^{0}<0$, where $X=\left[X^{i}\right],X^{i}\in F_{t}^{f,h},$ is the covariate vector for the specific number of features f and history length h, and $\theta \in R^{f\times h}$ and $\theta^{0}\in R$ are parameters. A probability outcome in $\left[0,1\right]$ can be obtained rather than the binary 0 or 1, based on the distance of θ·X to zero.	parallelSVM function, with the probability option, from the package parallelSVM (Rosiers, 2015)
Generalized linear model (GLM)	Generalizes the linear model for response variables with a non‐normal error distribution. Sine the response variable is binary, we use a binomial error distribution, which makes the GLM a logistic regression. The probability of MPB presence $P\left(I_{g,t+r}\right)$ is then modeled by $\frac{\exp\left(\theta \cdot X+\theta^{0}\right)}{1+\exp\left(\theta \cdot X+\theta^{0}\right)}$.	glm function of the package stats (R Core Team, 2018)
Generalized boosted (classification) model (GBM)	Reduces a loss function between the observed and predicted target values using Friedman's Gradient Boosting Machine (Ridgeway, 2006) on a certain number of classification trees.	gbm function of the package gbm (Ridgeway, 2006) using 10,000 trees
Naive Bayes network (NB)	Formed by one target node ($I_{g,t+r}$), linked to all covariates (Koller & Friedman, 2009) (Figure S3b). We use discrete variables for this and the following two Bayesian networks. We discretize the values of each non‐binary covariate into five equal levels.	package bnlearn (Scutari, 2010)
Chow‐Liu (CL)	A Bayesian network in the form of an undirected spanning tree of the variables that minimizes the *Kullback‐Leibler (KL) distance* (over all tree structures) from the actual distribution (Chow & Liu, 1968) (Figure S3a). Note that target node $I_{g,t}$ can be anywhere in this tree structure.	package bnlearn (Scutari, 2010)
Incremental association Markov blanket (IAMB)	A Bayesian network obtained by detecting Markov blankets with an attempt to avoid *false positives*, i.e., fault infestation predictions (Tsamardinos et al., 2003).	package bnlearn (Scutari, 2010)
k‐nearest neighbors (KNN)	A non‐parametric method that classifies the target variable of an instance in the test/validation dataset based on the classes (values) of the target variables of k other (training set) instances that share the most similar features – referred to as the neighbors (Altman, 1992). Similarity is often measured by the simple $l^{2}$‐norm $P\cdot P_{2}$. A probabilistic classification can be achieved based on the portion of neighbors who agree on the same class.	knn function with k=15 from the package class (Venables & Ripley, 2002)
(Artificial) neural network (NN)	A network of the so‐called *neurons* that change and then output the inputs the receive based on their activation function (Haykin, 1994). We train a neural network with one hidden layer with the number of nodes equal to half of the total number of used covariates, and the sigmoid activation function.	nn.train function of the package deepnet (Rong, 2014)
Mixed model (MM)	We construct a mixed model of all the previous ones in the form of a GLM of their outputs: $P\left(I_{g,t+r}\right)=\frac{\exp\left(\sum_{i=1}^{8}\theta^{i}P^{i}\left(I_{g,t+r}\right)+\theta^{0}\right)}{1+\exp\left(\sum_{i=1}^{8}\theta^{i}P^{i}\left(I_{g,t+r}\right)+\theta^{0}\right)}$, where $P^{1},\ldots ,P^{8}$ are the probabilities produced by models 1,…,8 above, and $\theta_{i=0}^{i^{8}}s$ are the parameters to be learned.	

### 2.7 Train and evaluation

**FIGURE 3:**
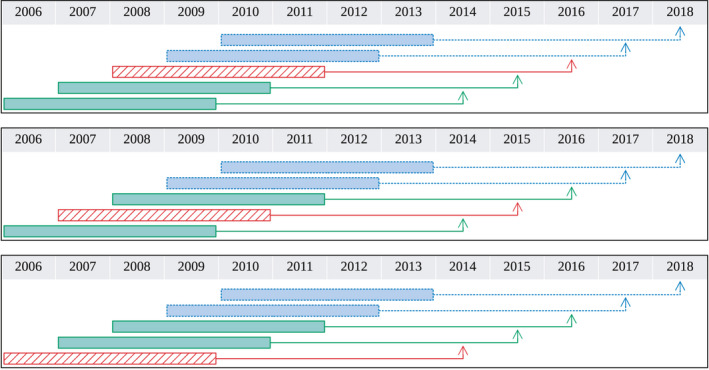
Dataset partition for cross‐validation. The boxes indicate which years the covariates are measured, and the arrows point to the year at which we predict infestation. Green solid lines represent the training set, whereas blue dashed lines represent the test set. Red hatched boxes represent which year in the training set was held out for cross‐validation. The top, middle and bottom represent the three different folds used in the cross‐validation process

For the training phase, we use cross‐validation on the train dataset. The data corresponding to each year is considered as a fold, and each time the predictive model is trained on all but one fold, and then evaluated on that held‐out fold (Figure 3). We evaluate each learner L based on the average *area under receiver operating characteristic curve (AUC)* (Metz, 1978; Bradley, 1997) of the models that L learned over the folds. Then for each future‐prediction‐length r and learner L, we find the number‐of‐covariates c and history‐length h that produced the highest cross‐validated AUC on the training dataset – call them $c^{*}$ and $h^{*}$. Next, based on the learner L, we learn a model on the whole training dataset $D_{\text{train}}^{c^{*},h^{*},r}$ and test it on the test dataset $D_{\text{test}}^{c^{*},h^{*},r}$ to obtain the AUC score $s_{L}$.

### 2.8 Estimating the ‘actual performance’

The test dataset is to represent that unavailable dataset that our final model will be applied to in practice. Hence, the performance of the learner over the test dataset – i.e., $s_{L}$ – may roughly be thought of its *actual performance*. To estimate this performance, we compare the following three AUC scores of the learner on the training dataset $D^{c^{*},h^{*},r}$: (i) $s_{L}^{\text{random}}$: obtained by randomly partitioning the train dataset into another train (70%) and test (30%), training the learner L on the train and testing it on the test; (ii) $s_{L}^{\text{average - fold}}$: the cross‐validated AUC explained above; (iii) $s_{L}^{\text{last - fold}}$: the AUC on the fold corresponding to the final year in the training dataset.

## 3 RESULTS

The mRMR method orders the covariates as in Table 4 (the phase covariate $O_{t}$ is excluded for r=7 as it is set to 3 in all data instances).

**TABLE 4:**
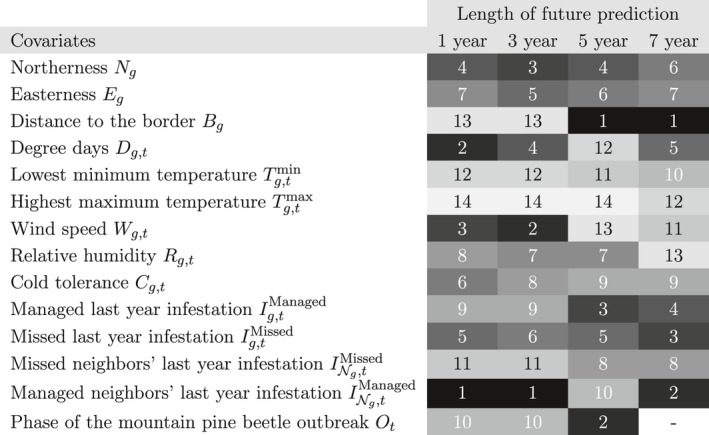
mRMR ranking results with respect to the target variable $I_{g,t+r}$. The numbers and cell shades represent the ordering of the covariates according to the mRMR method: 1 (black) is the covariate with the highest rank and 14 (lighter gray) is the covariate with the lowest rank

On the train dataset, and for r=1 and 3, most learners achieve their highest cross‐validated AUC when they use most of the covariates, e.g., $c^{*}=12$ (Table 5 – see also Figure S5 to S8 for the cross‐validated AUC of each learned model over all number‐of‐covariates c and history‐lengths h). This optimal number of features decreases as the prediction‐length r increases. For r=1,3,5, the cross‐validated AUC of NN increase with history length, and nearly the same holds with GBM and NB for r=1,3. However, the trend is often the opposite with GLM and roughly KNN. For r=7, the AUC of almost all models, except for NB, decreases with history‐length.

**TABLE 5:** Performance of the learners

length of future prediction (r)	Learners with $s_{L}^{\text{average}‐\text{fold}}\geq 0.8$	Learner with the highest AUC on the test dataset ($s_{L})$	$c^{*}$	$h^{*}$	AUC on the test dataset ($s_{L}$)
1 year	GBM, NN, MM	GBM	12	5	0.92
3 years	GBM, NB, NN, MM	GBM	14	5	0.88
5 years	GBM, KNN, MM	MM	5	2	0.86
7 years	KNN, MM	MM	4	1	0.84

On the test dataset, a GBM with 12 covariates and 5 years of history outperforms others in predicting future 1‐ and 3‐year infestations with AUC scores of 0.92 and 0.88 (Table 5). An MM with 5 covariates and 2 years of history and another with 4 covariates and 1 year of history, best predict future 5‐year (0.86 AUC) and 7‐year (0.84 AUC) infestations. Overall, and all prediction lengths (r) considered, GBM is ranked first on the test dataset (Table S1), and MM and NB are the next best predictors.

**FIGURE 4:**
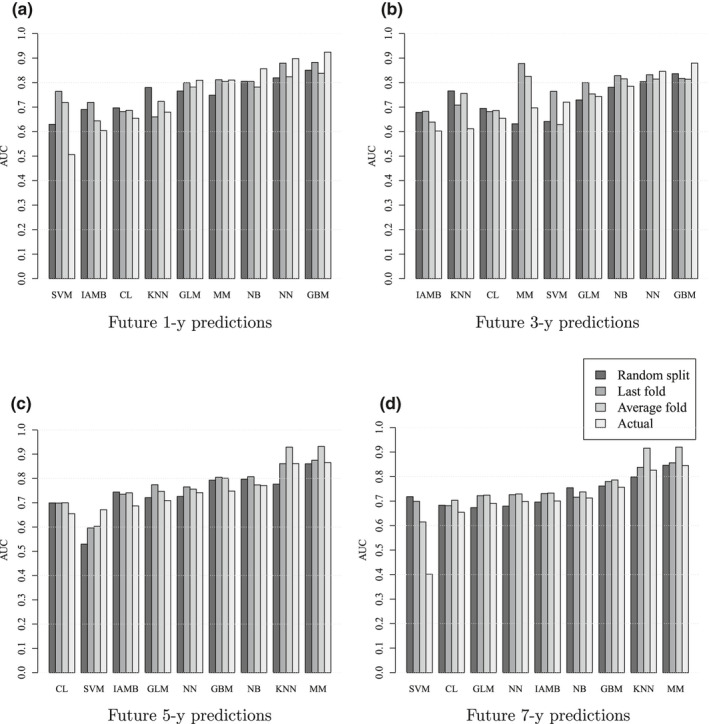
The actual AUC score on predicting infestations at years 2017 and 2018, and its estimations based on different train‐test partitioning. White, light gray, dark gray and black are the AUC scores on the test dataset (“actual,” $s_{L}$), cross‐validated AUC on the train dataset (“average fold,” $s_{L}^{\text{average}‐\text{fold}}$), AUC on the last year of the train dataset (“last fold,” $s_{L}^{\text{last}‐\text{fold}}$), and AUC on the test dataset obtained from a random partitioning of the training dataset into another train and test (“random split,” $s_{L}^{\text{random}}$). The learners are those listed in Table 3 and are ordered from right to left on the x‐axis based on their scores on the test dataset – i.e., $s_{L}$ (the white bars). (a)–(d) are future 1‐, 3‐, 5‐, and 7‐year predictions. The estimated AUC based on the last‐fold partitioning best matches the actual AUC for the top‐two learners (except for GBM at future 3‐year predictions)

**FIGURE 5:**
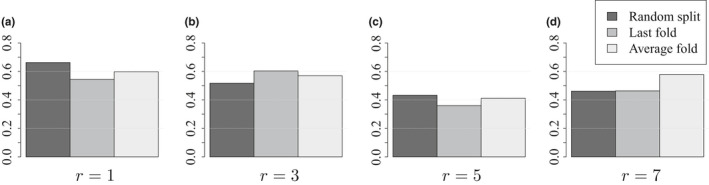
Absolute estimation error of the AUC score on years 2017 and 2018, accumulated over the learners. Light gray, dark gray and black are $\sum_{L}\left|S_{L}^{\text{average - fold}}‐S_{L}\right|$, $\sum_{L}\left|S_{L}^{\text{last - fold}}‐S_{L}\right|$, and $\sum_{L}\left|S_{L}^{\text{random}}‐S_{L}\right|$. (a)–(d) are for future 1‐, 3‐, 5‐, and 7‐year predictions. Overall, last‐fold partitioning best estimates the actual AUC score over all learners

The AUC score of each learner on the test dataset together with its three estimations are shown in Figure 4. For almost any future prediction‐length r, the score $s_{L}$ of the top‐two learners on the test dataset is best estimated by $s_{L}^{\text{last - fold}}$. Moreover, the absolute AUC estimation error of each estimator and over all learners – i.e., $\sum_{L}\left|{\hat{s}}_{L}‐s_{L}\right|$, where ${\hat{s}}_{L}\in \left\{s_{L}^{\text{random}},s_{L}^{\text{last - fold}},s_{L}^{\text{average - fold}}\right\}$ – is always lowest for the last‐fold, except for r=3, where the random‐fold has the lowest error (Figure 5).

**FIGURE 6:**
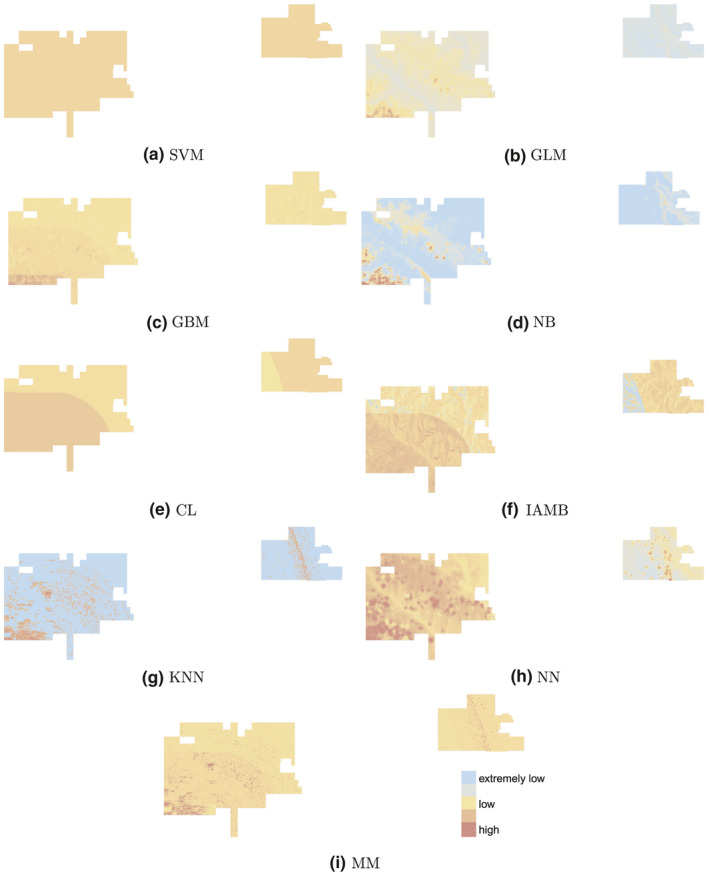
Comparison of infestation maps of year 2018 predicted by each of the learners using data prior to year 2013 (future 5‐year prediction). Each learner assigns an infestation probability to every pixel which is represented on a log scale from extremely low (blue) to high (red)

**FIGURE 7:**
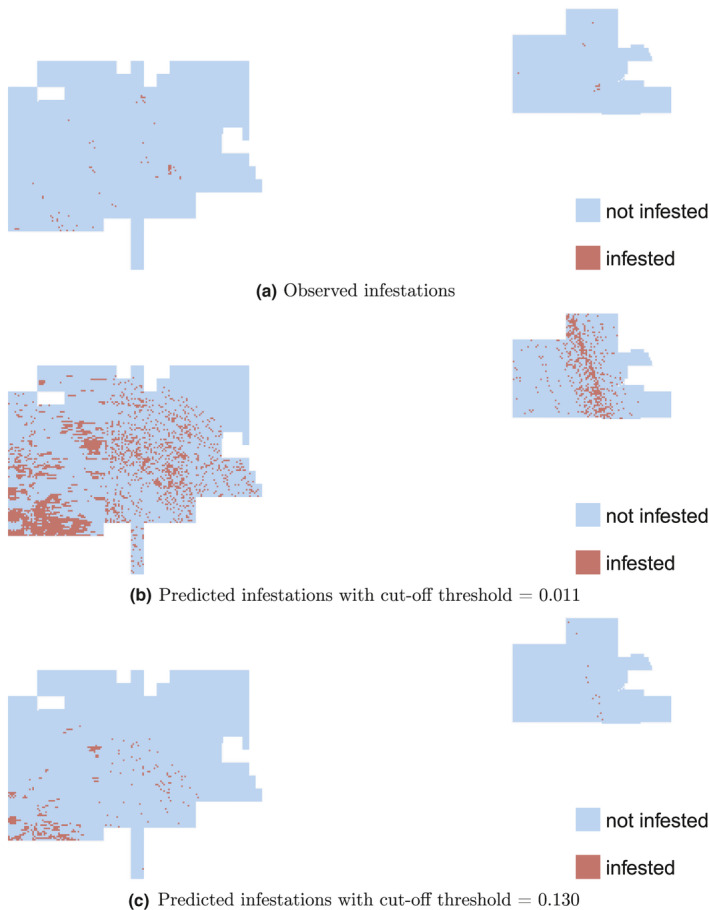
(a) Observed infestations, (b) predicted infestations using a cut‐off threshold of 0.011, and (c) predicted infestations using a cut‐off threshold of 0.130, for the year 2018 (future 5‐year infestations). The infestation probabilities are calculated using the learner with the highest AUC (i.e., MM) on predicting future 5‐year infestations on the test dataset (Figure 6i). Then the binary predictions in (b) are generated using the optimal cut‐off threshold derived from Youden's index, which maximizes the summation of sensitivity and specificity. The binary predictions in (c) are generated similarly to (b) but when specificity is weighted 10 times more than sensitivity. As the cut‐off threshold increases, fewer pixels are predicted as infested

Using the data prior to and including 2013, most learners predict the south‐west border and some areas in the center of the two portions of the park as infested at year 2018 (Figure 6). The actual infestation map at year 2018 confirms these infestations (Figure 7a). For management purposes, the probabilistic infestation maps can be turned into binary infestation maps using a cut‐off threshold. The highest‐scoring learner at predicting future 5‐year infestations, i.e., MM, predicts more pixels than observed as infested when Youden's optimal cut‐off threshold is used (Youden, 1950) (Figure 7b). This threshold maximizes the summation of *sensitivity* and *specificity* (Metz, 1978). If we put more weight on specificity, say 10 times more than sensitivity, then the number of pixels that are predicted infected will be closer to that of the observed (Figure 7c).

## 4 DISCUSSION

The spectacular results of machine learning in many areas (Makridakis et al., 2018; Olden et al., 2008) makes it a tempting choice for predicting future infestations. Achieving accurate results, however, require thoughtful use and implementation of the even standard models (Olden et al., 2008) as this often requires identifying the most effective base learner, as well as the features to use (here, which covariates, over what specific history length). Also, one needs to properly evaluate the models to avoid misleading performance evaluations (Mouton et al., 2010), as unfortunately often practiced. We have addressed these problems for a controlled mountain pine beetle outbreak in the Cypress Hills area, and trained two GBMs predicting future 1‐ and 3‐year infestations with 92% and 88% AUC, and two novel mixed models predicting future 5‐ and 7‐year infestations with 86% and 84% AUC, respectively.

The trained models seem to greatly outperform the existing models in the literature. For example, the GBM scores 88% AUC on predicting future 3‐year infestations, whereas the logistic regression model in (Aukema et al., 2008) scores 30.5% on accuracy with zero false negatives.

One common approach to predicting future infestations, say 50‐year, using temporal environmental covariates such as climate variables is to first predict future values of those covariates, then use those values to predict future infestations (Broennimann & Guisan, 2008). Two separate models are used for these two phases. For example, to predict infestations at year 2050 based on temperature and humidity at year 2000, first, a model A is used to predict temperature and humidity at year 2050 and then a model B is used to predict infestations at 2050 based on the predicted temperature and humidity at 2050. However, more accurate results may be achieved by predicting future infestations directly based on the current values of the temporal covariates by a single model C. The reason is that infestations at year 2050 may not depend on the exact values of temperature and humidity at 2050, but a specific function of them and perhaps other variables, which may be better estimated directly from temperature and humidity at year 2000. This particularly holds if model C is complex enough to implicitly perform what models A and B can do consecutively.

### 4.1 mRMR ranking

Although unfamiliar to many ecologists (but see Hejazi & Cai, 2009; Li et al., 2018), the mRMR ranking method has potential to reduce model complexity by identifying the most relevant set of features in a dataset. Managed neighbors’ last year infestation $I_{N_{g},t}^{\text{Managed}}$ is ranked first by mRMR for predicting future 1‐ and 3‐year infestations. This means that managed last‐year infestations at the neighboring pixels has the greatest correlation with the presence of short‐term future infestation. This is in line with studies reporting strong spatial and temporal dependencies in small scales (Aukema et al., 2008; Preisler et al., 2012). Even though the infestations at the neighboring pixels are managed, they are still the most informative covariate for future infestations, perhaps because they are the best indicator of suitable MPB habitats. However, for intermediate‐term predictions – i.e., 5 and 7 years – distance to infested border $B_{g}$ is a more‐informative covariate, because future 5‐year infestation patterns will not be similar to how they were last year and mainly influenced by the source of the infestation.

For future 1‐year infestations, the second ranked covariate, degree days $D_{g,t}$, has the greatest correlation with the target $I_{g,t+1}$ after removing its correlation with $I_{N_{g},t}^{\text{Managed}}$. However, it cannot be inferred that models trained with these two covariates outperform those trained with any other two covariates, because not every model suffers from correlated covariates, but may even benefit; namely, correlation does not imply dependence but could be simply some residual information. Similarly, wind speed $W_{g,t}$ is the second most‐informative covariate in predicting future 3‐year infestations but is covered by other covariates or insufficiently correlated with the target variable for future 5‐ and 7‐year infestations. Note that the mRMR ranking differs from rankings based on the maximum likelihood estimate of the covariates or standard errors of the covariates as they do not incorporate the *minimum redundancy* Sambaraju et al. (2012). This may explain the inconsistency with Aukema et al. (2008) that does not find degree days a significant predictor.

Ranked poorly in all prediction‐lengths, temperature covariates $T_{g,t}^{\text{min}}$ and $T_{g,t}^{\text{max}}$ almost do not increase our knowledge about future infestations, beyond what the other covariates provide. However, this does not imply that they are least correlated with the target variable $I_{g,t+r}$ but that their information is better covered by the covariates that appear early in the ranking.

Interestingly, the simplest covariate, outbreak phase $O_{t}$, is the most informative in predicting future 5‐year infestations, after $B_{g}$. That is, the current phase of the outbreak has the highest correlation with the presence of infestation over all pixels, after removing its correlation with $B_{g}$. However, almost none of the models immediately benefit from this covariate after it is added to $B_{g}$ during the training phase. In a similar fashion, (Kunegel‐Lion & Lewis, 2020) found that the predicting future 1‐year infestations does depend on the outbreak phase.

### 4.2 Number of optimal covariates

The number of features resulting in the highest cross‐validated AUC on the training dataset generally decreases as the prediction‐length increases. For r=1 and 3, the best predictors use almost all of the available covariates and history‐length, confirming the success of the all‐inclusive model in (Aukema et al., 2008). However, for r=7, the top predictors use only one year of history length, and the best predictor, MM, uses four covariates. Interestingly, this means that if we know the distance of a given pixel to the infested border and last year infestation status of the pixel and its neighbors, then we can predict whether the pixel will be infested in the future seven years, with 0.84 AUC. None of the climate covariates, nor the geographic covariates northerness and easterness are required. Studies on other species (de la Fuente et al., 2018) also found that information on previous infestations without using environmental covariates is sufficient to make accurate predictions. Our results, however, do not contrast studies claiming a strong relationship between climate covariates and concurrent or near‐future infestations (Preisler et al., 2012).

We also observe that some learners, such as GBM, generally tend to use more covariates. One may, therefore, try to provide as many covariates and history‐length as possible when using such learners, especially for short‐term future predictions as in (Aukema et al., 2008).

### 4.3 History‐length selection

Unlike studies that decide a priori on the amount of lag for the covariates (Aukema et al., 2008), we investigate the lag time that results in the highest performance of the learners using the data. The prediction accuracy of NN, GBM, and NB increases as we increase the history‐length of their covariates for future 1‐, 3‐, and roughly 5‐year infestations. We refer to models with this property as *history‐friendly* since increasing the history length does not lead them to overfit, and hence, one may freely do so with the hope of achieving a more accurate model. Interestingly, these three models are highly nonlinear, and the linear model SVM, and even generalized linear model GLM, do not exhibit this characteristic for this specific task. Hence, some degree of non‐linearity is required for being history‐friendly, at least on our dataset. Likewise, MM is not history friendly, perhaps partly because it is a GLM‐combination of the other models. On the other hand, the failure of KNN in exploiting history implies that providing history leads to instances that are similar to the instance in question but have a different infestation value, where similarity is with respect to geometric distance in the feature space.

### 4.4 Model comparison

Overall, the simple boosted decision tree outperforms all other learners, including the complex NN, in short‐term predictions, and performs fairly well for long‐term predictions.

The second‐best learner is the most complicated, MM, which outperforms others in predicting intermediate‐term infestations. We do expect MM to excel at the training phase, but not necessarily at the test, due to the possibility of overfitting the training dataset. This is particularly true for predicting future 3‐year infestations, as MM is the best predictor at train but ranked 6th during the test.

The third‐best predictor is NB, which has a unique advantage over all other models that it can still predict infestation when the values of one or more of the covariates are missing. Thus, if missing values is a concern, perhaps the best model is NB.

KNN performs well only in predicting future 5‐ and 7‐years. Hence, by directly comparing the instance in question with those that had similar features in the past years, we can accurately predict intermediate‐term infestations. The same does not hold for 1‐year predictions, implying the existence of pixels with similar features, yet different infestation statuses.

The one‐layer neural network is the second‐best predictor in predicting future 1‐ and 3‐year infestations. Therefore, both the simple GBM and complicated NN are capable of accurately predicting short‐term future infestations. However, due to its simplicity, one may subjectively find GBM more reliable than the neural network, and hence, pick it as the best predictor. The incapability of NN in predicting the intermediate‐term future may imply the need for a more sophisticated NN structure.

The poor performance of SVM and GLM is an indicator of the dataset not being linearly separable, and also a sign of caution for applying the commonly used GLM for prediction purposes.

Given the success of NB, the failure of the searching‐algorithm IAMB implies that ‘the right’ Markov blankets are not easy to find. Similarly, the failure of CL implies that tree structures with the minimum KL difference are not promising predictors for our dataset.

### 4.5 Model evaluation

How do we decide which learner to use for predicting a real‐world process in the future? We never know the actual performance of a trained model in predicting the future, unless we wait for the future to arrive We can only estimate the actual performance. This is typically done by randomly partitioning the available dataset into training and test datasets, training the model on the training dataset, and taking its score on the test dataset as an estimation of its actual performance (Broennimann & Guisan, 2008). One essential contribution of this paper is to show that this random split may lead to models that perform well in simulations, but poorly in practice, or *vice‐versa*. For example, compared to its actual performance on the held‐out test dataset, KNN performs 10% higher at AUC under the evaluation provided by a random split. The same holds for any other partitioning, where the train and test include instances at the same year (de la Fuente et al., 2018).

A random split is plausible, provided that the instances are independent and identically distributed (iid). However, the data in a temporal process is not iid, as data at time t+1 depends on data at time t; namely, future instances depend on current ones. This conclusion agrees with (Bahn & McGill, 2013), which found that the predictive accuracy decreases with increases in the independence between training and test sets. For the same reason, performing cross‐validation may not well represent the actual performance either.

To obtain a proper estimation, we need to mimic how the model will be used in practice. Namely, in a real‐world scenario, the data from the future is not available, and hence, the model can never be trained on it. So instances from later years must not be included in the training dataset and should form the validation. We call this a *year‐based* or, in general, a *temporal split* of the dataset. Although this type of partitioning has been appropriately implemented in some studies (Aukema et al., 2008; Meentemeyer et al., 2011), it has not been addressed in detail in the literature as most data in machine learning are iid, and hence, do not encounter these challenges. In our MPB case study, the evaluations obtained from a year‐based split best estimate the performance of the top models. Nevertheless, the random split does not always result in a worse estimation.

**FIGURE 8:**
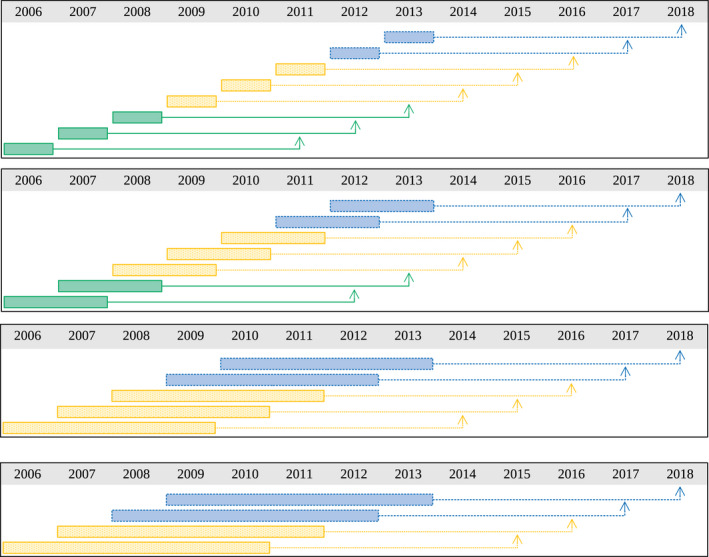
Dataset partition for r=5 years in the future, honoring the “temporal gap”. The figure differs from Figure 2 only by coloring the “gap instances” as yellow, to indicate that they should not be used during the training nor the validation phases – which significantly decreases the size of the training dataset. In particular, the bottom two subfigures corresponding to *h* = 4 and *h* = 5 result in zero training instances

Indeed, a proper estimation of the actual performance requires further restrictions on the training dataset. If we were in 2013 and wanted to predict the year 2018, the information for 2018 would not be available, nor would any information for years 2014–2017. Hence, the training data (for training this “2013‐model‐for‐predicting‐2018”) should not include any of the instances whose target variables are at years 2014, 2015, 2016, and 2017. They should not be used during the validation phase either. That is, there should be a “temporal gap” between the training and testing datasets (Ramazi et al., 2021). More generally, when predicting year t+r from year t, all data instances with target variables at and prior to year t form the training dataset, the data instance whose target variable is at year t+r forms the testing dataset, and the instances in between (i.e., in years t+1,..., t+r‐1), form the gap and may not be used. Such partitioning, however, may result in few, or even zero, training instances. For example, in the case of r=5 and h=1 in our case study, all instances whose target variable is at a year later than 2013 should be eliminated from the training dataset (Figure 8). In case of r=5 and h=4 or h=5, this results in zero training instances. We have, therefore, not used this restrictive yet appropriate partitioning. However, future studies may investigate this issue for the case of r=1 and r=3.

### 4.6 Future work

Further studies are required to find conditions under which learners predict more accurately on a randomly‐obtained test dataset than a year‐based one. It is also of great interest to examine the newly introduced mixed model for prediction lengths longer than seven years. One may try to further explore this model by constructing a neural‐network mixture of the other models instead of the GLM mixture.

## CONFLICT OF INTEREST

The authors declare no conflict of interest.

## AUTHOR CONTRIBUTIONS

**Pouria Ramazi:** Conceptualization (equal); formal analysis (lead); investigation (equal); methodology (lead); software (equal); validation (equal); visualization (equal); writing–original draft (equal); writing–review and editing (equal). **Mélodie Kunegel‐Lion:** conceptualization (equal); data curation (equal); formal analysis (equal); investigation (equal); methodology (lead); software (equal); validation (equal); visualization (equal); writing–original draft (equal); writing–review and editing (equal). **Russell Greiner:** Conceptualization (equal); funding acquisition (equal); investigation (equal); methodology (equal); supervision (equal); validation (equal); writing–original draft (equal); writing–review and editing (equal). **Mark A. Lewis:** Conceptualization (equal); funding acquisition (equal); investigation (equal); methodology (equal); supervision (equal); validation (equal); writing–original draft (equal); writing–review and editing (equal).

All authors conceived the ideas, interpreted the results and drafted the manuscript. P.R. developed the methods and under‐took the analysis. All authors gave final approval for publication.

## Supporting information

Supporting Information

## Data Availability

The dataset analyzed in the current study is described in (Kunegel‐Lion et al., 2020a) and avail able from Dryad repository (https://doi.org/10.5061/dryad.70rxwdbt9) (Kunegel‐Lion et al., 2020b).
