# Supplementary material for: Predicting insect outbreaks using machine learning: A mountain pine beetle case study
Source: Ecol Evol. 2021 Sep 12;11(19):13014–28. doi: 10.1002/ece3.7921 (PMC8495826; doi:10.1002/ece3.7921)
Supplement: Supplementary file 1 — Supporting Information [file ECE3-11-13014-s001.pdf]

Running title: Predicting Insect Outbreaks Using Machine Learning

Supplementary Material for Predicting Insect Outbreaks Using Machine Learning: A Mountain Pine Beetle Case Study

Pouria Ramazi[1,2,*], **Mélodie Kunegel-Lion**[3], **Russell Greiner**[2,4], **& Mark A. Lewis**[1,3]

1. *Department of Mathematical and Statistical Sciences, University of Alberta, Edmonton, AB, T6G 2G1 Canada*
2. *Department of Computing Science, University of Alberta, Edmonton, AB, T6G 2E8 Canada*
3. *Department of Biological Sciences, University of Alberta, Edmonton, AB, T6G 2E9 Canada*
4. *Alberta Machine Intelligence Institute, Edmonton, AB, T5J 3B1 Canada*

*Correspondence author. p.ramazi@gmail.com

Materials and methods

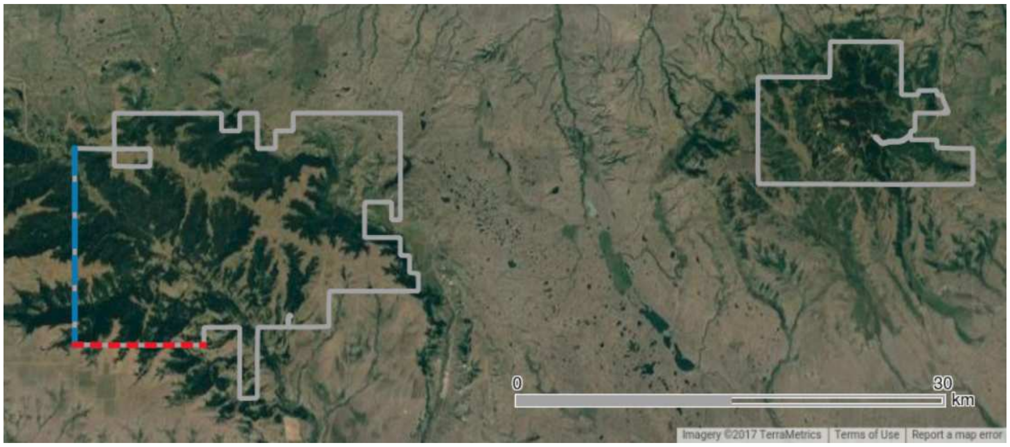

Figure 1 – **Cypress Hills park boundaries in Saskatchewan** (Based on Kunegel-Lion *et al.* (2019)). The grey, red and blue lines represent the borders of the park, the border close to outside infestations in the South and the border with Alberta, respectively.

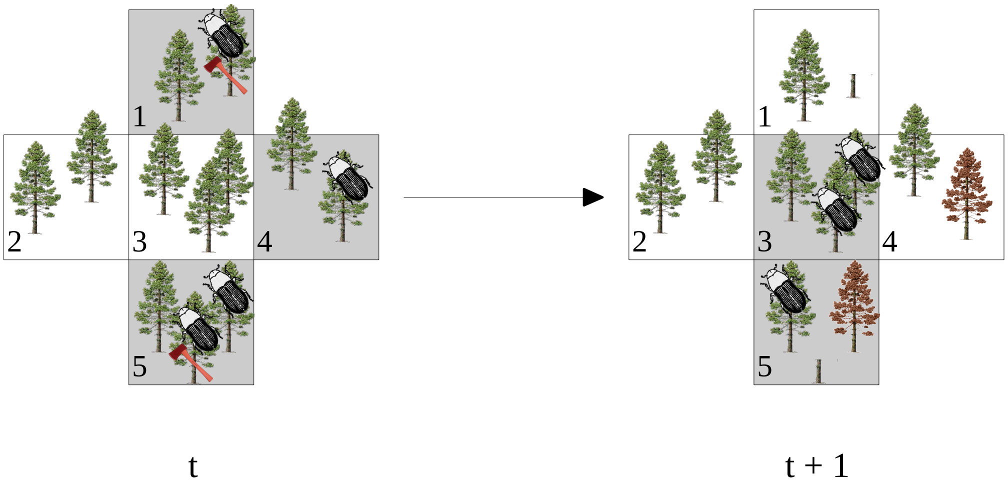

Figure 2 – **Infestation status.** Gray and white are used to indicate the presence and absence of infestation in a pixel at a given year. (Left) none of the trees in pixel 3 were infested at year t ($I_{3,t} = 0$); however, (right) at least one tree in this pixel was infested at year $t+1$ ($I_{3,t+1} = 1$). All infested trees in pixel 1 that were infested at year t were managed at the same year ($I_{1,t}^{\text{Managed}} = 1$, $I_{1,t}^{\text{Missed}} = 0$), there were no infested trees in pixel 2 at year t ($I_{2,t}^{\text{Managed}} = 0$, $I_{2,t}^{\text{Missed}} = 0$), all infested trees in pixel 4 that were infested at year t, were missed at the same year, and hence, turned red in the following year ($I_{4,t}^{\text{Managed}} = 0$, $I_{4,t}^{\text{Missed}} = 1$), some infested trees were missed and some were managed in pixel 5 ($I_{5,t}^{\text{Managed}} = 1$, $I_{5,t}^{\text{Missed}} = 1$). Missed and managed neighbors' last year infestation for pixel 3 at year t are, thus, $I_{\mathcal{N}_3,t}^{\text{Missed}} = I_{\mathcal{N}_3,t}^{\text{Managed}} = \frac{1}{2} + \frac{1}{2}$, presuming that $\mathcal{N}_3^2 = \mathcal{N}_3^3 = \emptyset$. The Figure is adapted from (Ramazi *et al.*, under review).

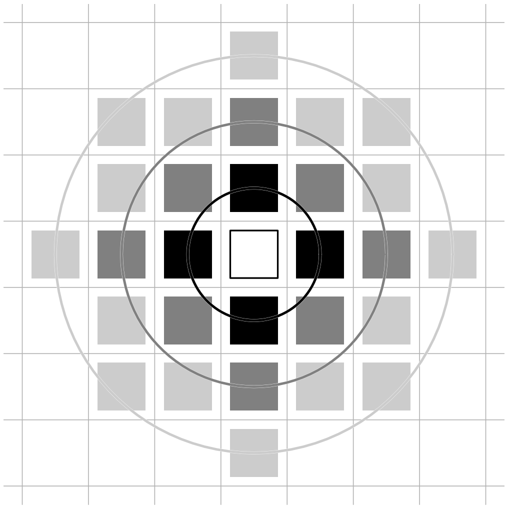

Figure 3 – **Representation of the neighbouring pixels. Center white:** g, the focus pixel, weighted 0 in the equation defining $I^{\text{Missed}}_{\mathcal{N}_g,t-1}$, **black:** $\mathcal{N}^1_g$, the 4 adjacent pixels within radius 1, each weighted $\frac{1}{2}$, **medium grey:** $\mathcal{N}^2_g$, the next 8 adjacent pixels within radius 2, each weighted $\frac{1}{4}$, **light grey:** the next 16 adjacent pixels within radius 3, each weighted $\frac{1}{8}$.

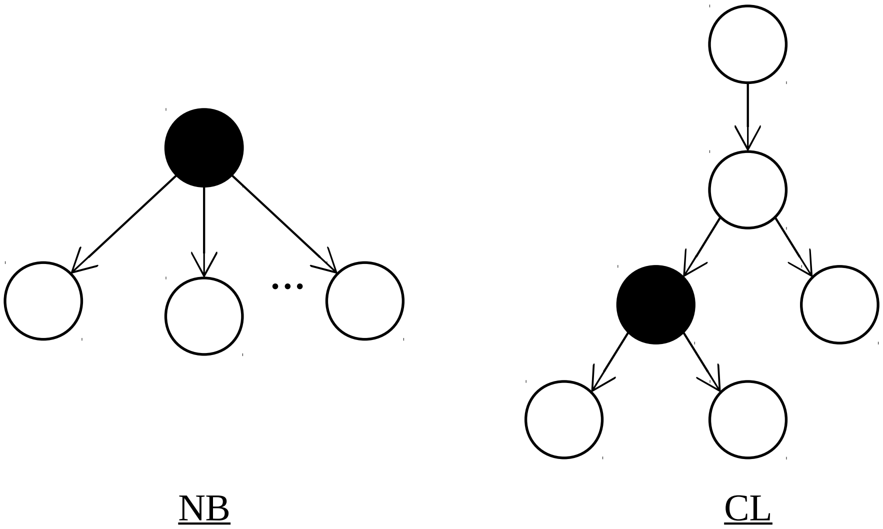

Figure 4 – **Structure of the Bayesian networks CL: Chow-Liu, NB: naive Bayesian.** Black and white circles represent, respectively, the target $I_{g,t}$ and its covariates.

mRMR ranking results

$$I_{g,t+1}: \; I^{\text{Managed}}_{\mathcal{N}_g,t} \succ D_{g,t} \succ W_{g,t} \succ N_g \succ I^{\text{Missed}}_{g,t} \succ C_{g,t} \succ E_g \succ R_{g,t} \succ I^{\text{Managed}}_{g,t} \succ O_t \succ I^{\text{Missed}}_{\mathcal{N}_g,t} \succ T^{\min}_{g,t} \succ B_g \succ T^{\max}_{g,t}, \quad (1)$$

$$I_{g,t+3}: \; I^{\text{Managed}}_{\mathcal{N}_g,t} \succ W_{g,t} \succ N_g \succ D_{g,t} \succ E_g \succ I^{\text{Missed}}_{g,t} \succ R_{g,t} \succ C_{g,t} \succ I^{\text{Managed}}_{g,t} \succ O_t \succ I^{\text{Missed}}_{\mathcal{N}_g,t} \succ T^{\min}_{g,t} \succ B_g \succ T^{\max}_{g,t}, \quad (2)$$

$$I_{g,t+5}: \; B_g \succ O_t \succ I^{\text{Managed}}_{g,t} \succ N_g \succ I^{\text{Missed}}_{g,t} \succ E_g \succ R_{g,t} \succ I^{\text{Missed}}_{\mathcal{N}_g,t} \succ C_{g,t} \succ I^{\text{Managed}}_{\mathcal{N}_g,t} \succ T^{\min}_{g,t} \succ D_{g,t} \succ W_{g,t} \succ T^{\max}_{g,t}, \quad (3)$$

$$I_{g,t+7}: \; B_g \succ I^{\text{Managed}}_{\mathcal{N}_g,t} \succ I^{\text{Missed}}_{g,t} \succ I^{\text{Managed}}_{g,t} \succ D_{g,t} \succ N_g \succ E_g \succ I^{\text{Missed}}_{\mathcal{N}_g,t} \succ C_{g,t} \succ T^{\min}_{g,t} \succ W_{g,t} \succ T^{\max}_{g,t} \succ R_{g,t}. \quad (4)$$

14 AUC results over different number of covariates and history lengths

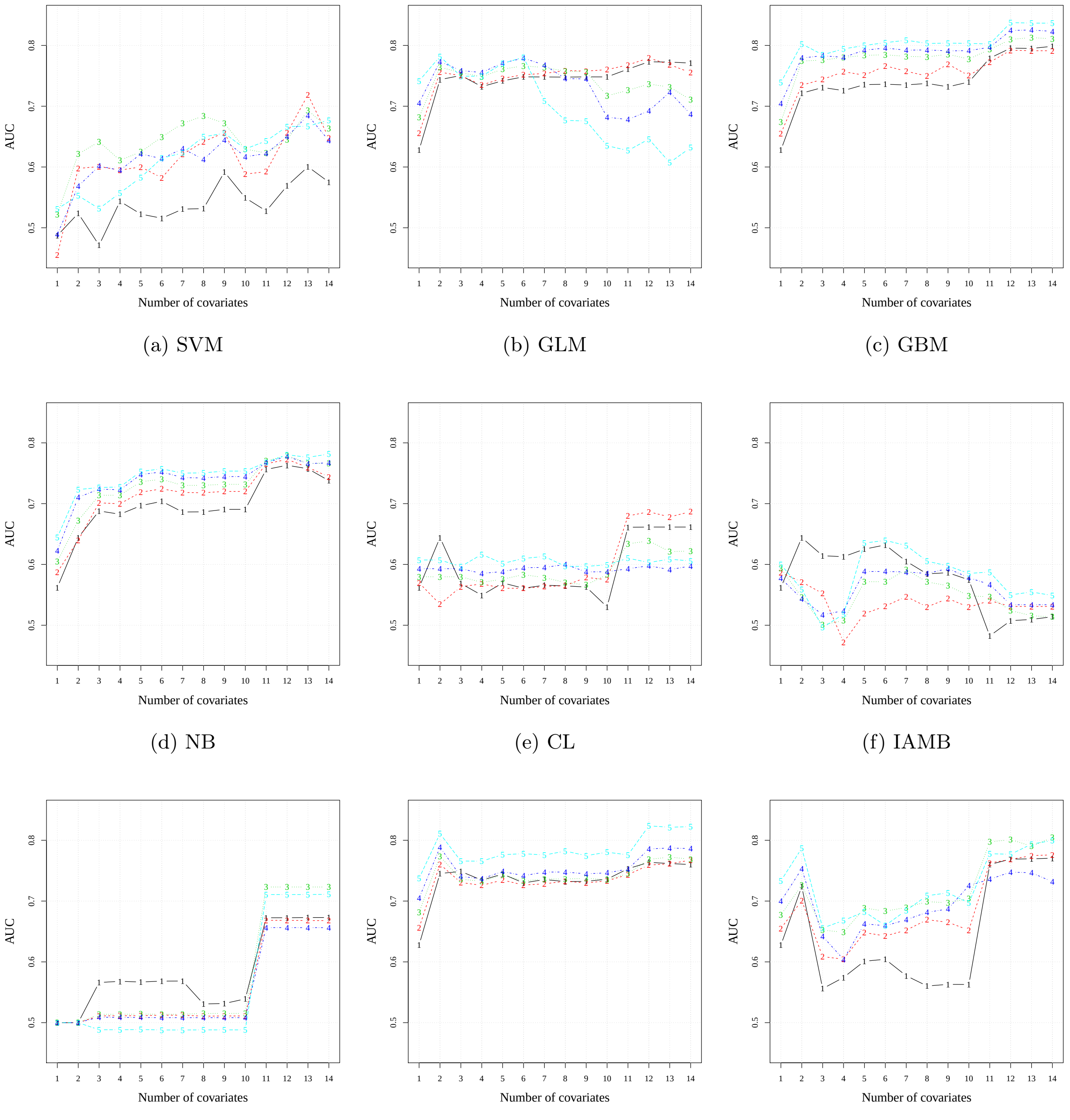

(a) SVM (b) GLM (c) GBM

(d) NB (e) CL (f) IAMB

(g) KNN (h) NN (i) MM

Figure 5 – **Future 1 year prediction.**

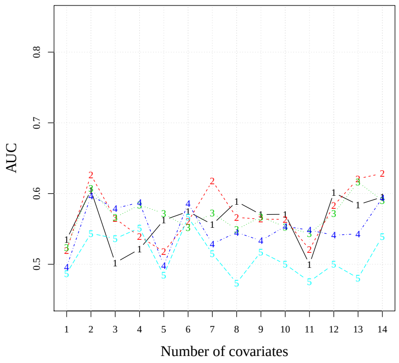

(a) SVM

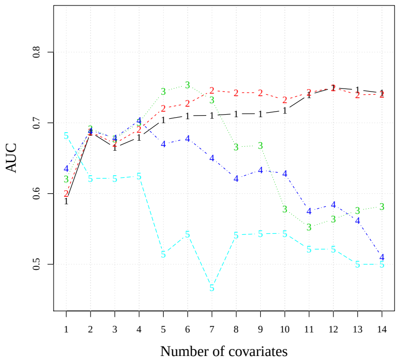

(b) GLM

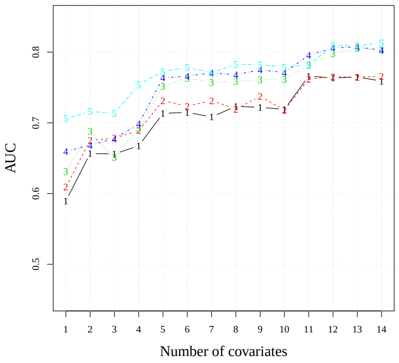

(c) GBM

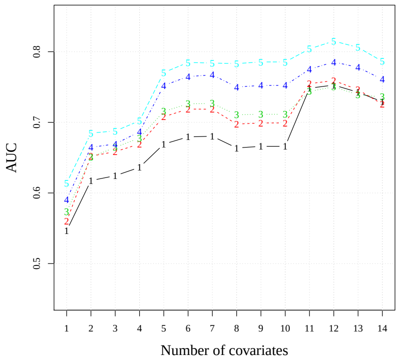

(d) NB

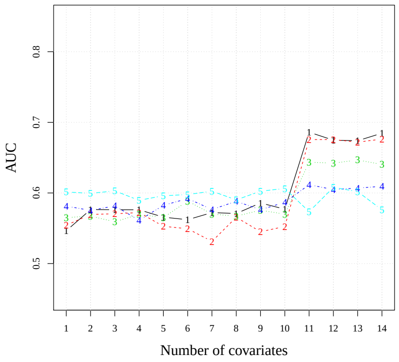

(e) CL

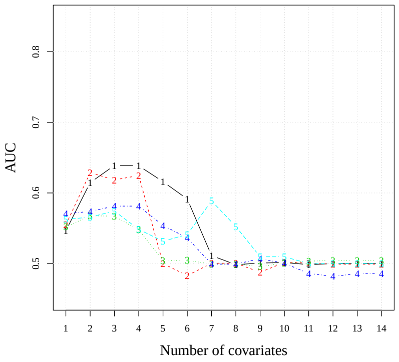

(f) IAMB

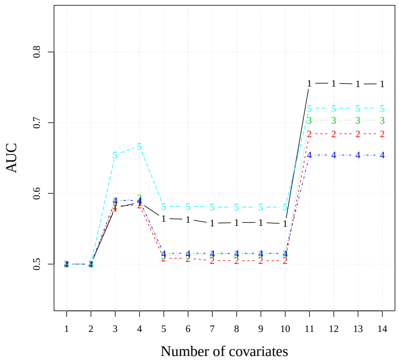

(g) KNN

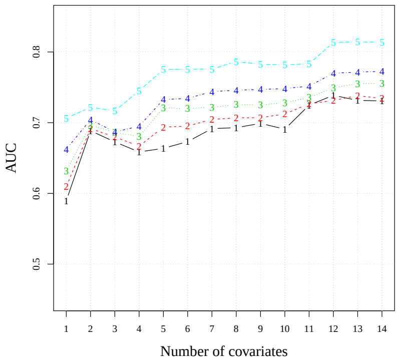

(h) NN

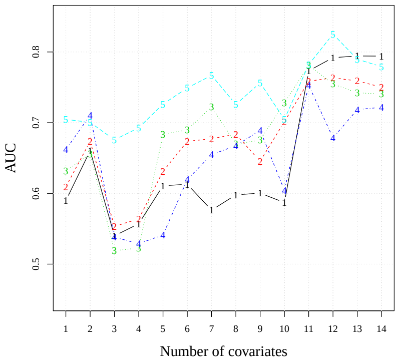

(i) MM

Figure 6 – **Future 3 years prediction.**

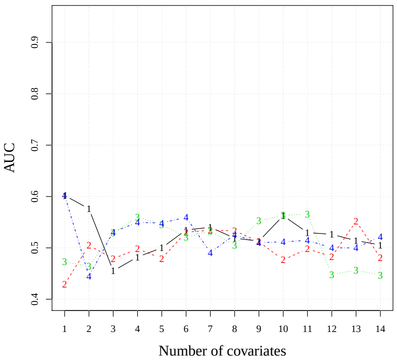

(a) SVM

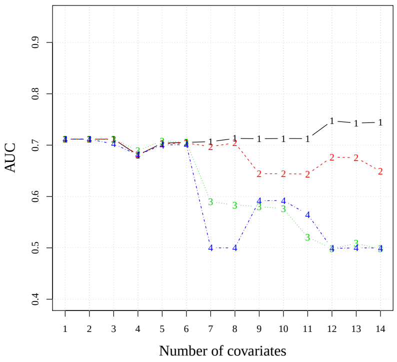

(b) GLM

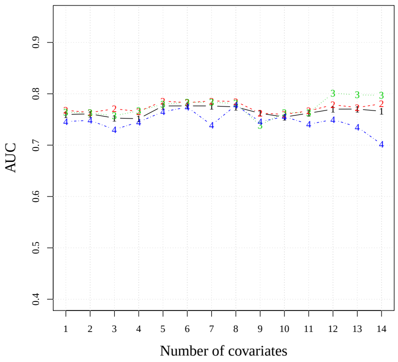

(c) GBM

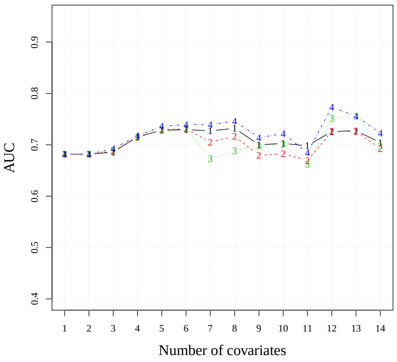

(d) NB

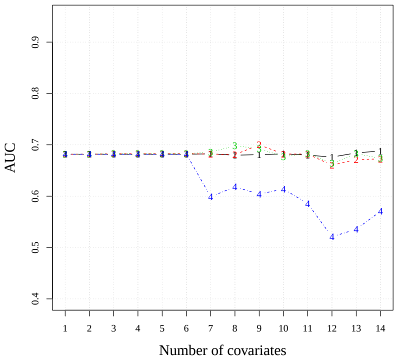

(e) CL

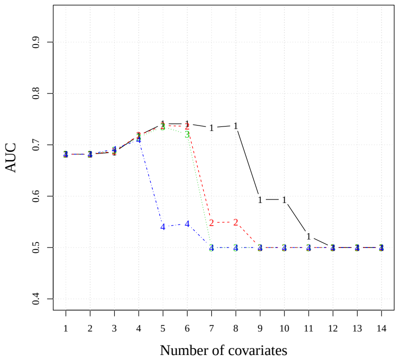

(f) IAMB

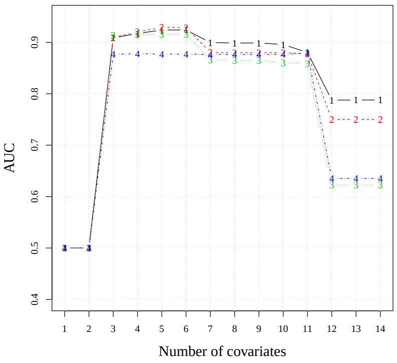

(g) KNN

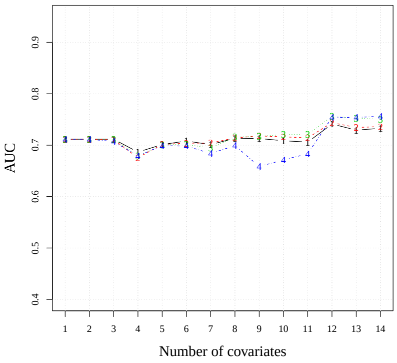

(h) NN

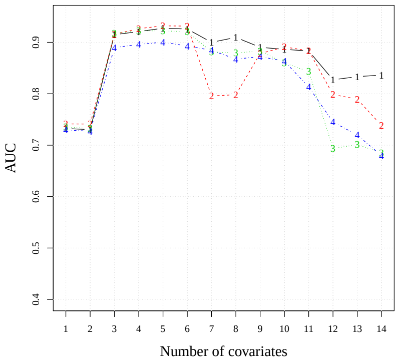

(i) MM

Figure 7 – **Future 5 years prediction.**

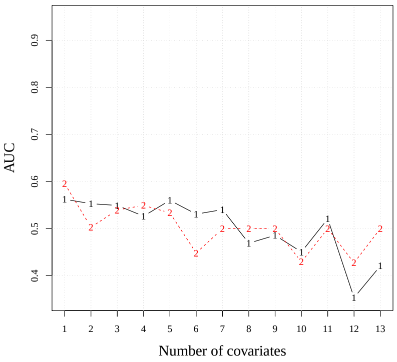

(a) SVM

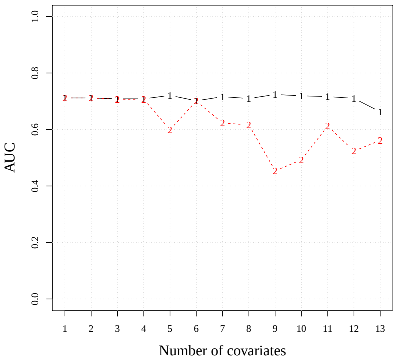

(b) GLM

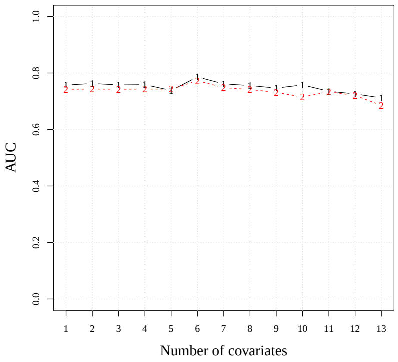

(c) GBM

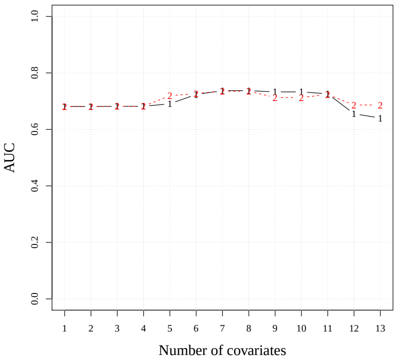

(d) NB

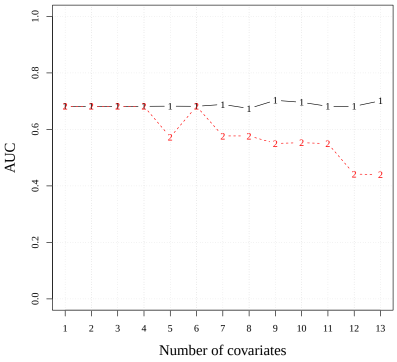

(e) CL

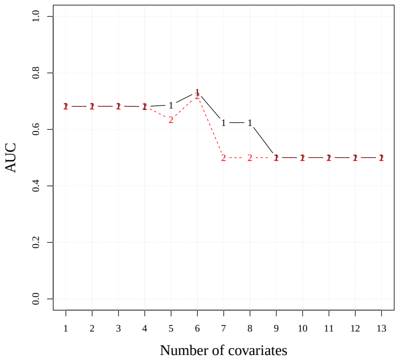

(f) IAMB

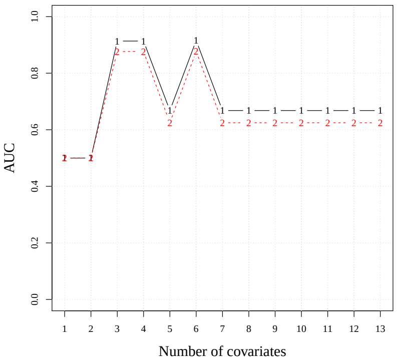

(g) KNN

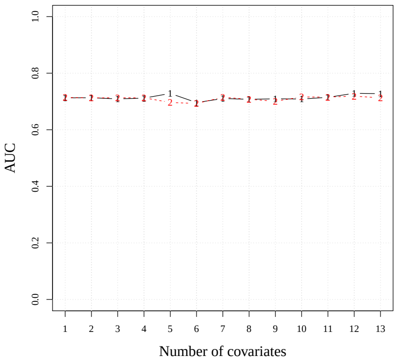

(h) NN

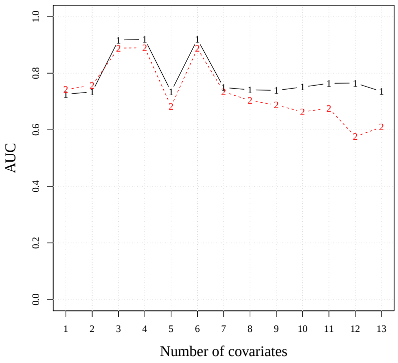

(i) MM

Figure 8 – **Future 7 years prediction.**

Overall ranking of the learners

Table 1 – Overall ranking of the learners based on their rankings in predicting different lengths of future prediction on the test dataset comprising instances from years 2017 and 2018.

**Length of future prediction (r)**	**SVM**	**CL**	**IAMB**	**GLM**	**NN**	**KNN**	**NB**	**MM**	**GBM**
1 year	9	7	8	5	2	6	3	4	1
3 years	5	7	9	4	2	8	3	6	1
5 years	9	8	7	6	5	2	3	1	4
7 years	8	9	5	7	6	2	4	1	3
average	8	7.75	7.25	5.5	5	4.5	3.25	3	2.5
**Overall ranking**	9	8	7	6	5	4	3	2	1

16 Predicting the infestation map of year 2023

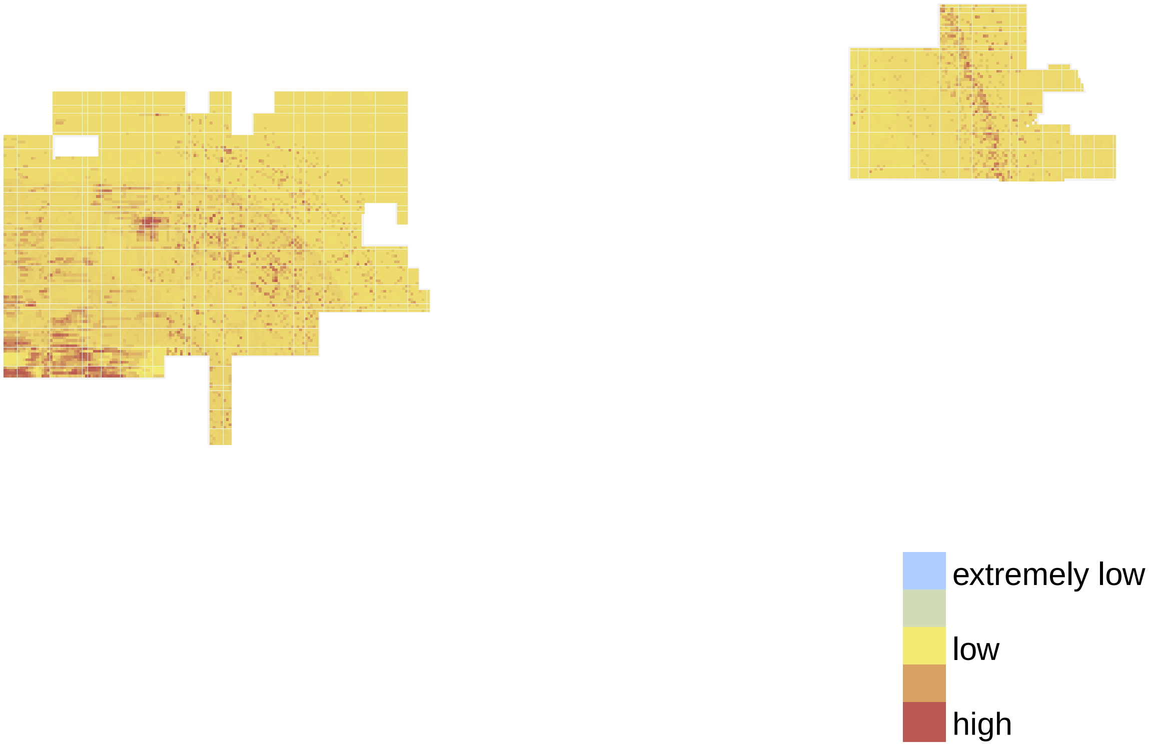

Figure 9 – **Infestation map.** Prediction of the infestation map of year 2023 using data prior to year 2019 (future 5-y infestations) by the best predictor on the test dataset – *i.e.*, MM. Most locations have a low infestation probability (yellow) and the remaining locations have a medium to high infestation probability (orange to red).

Results for when current (instead of past) managed infestations are used

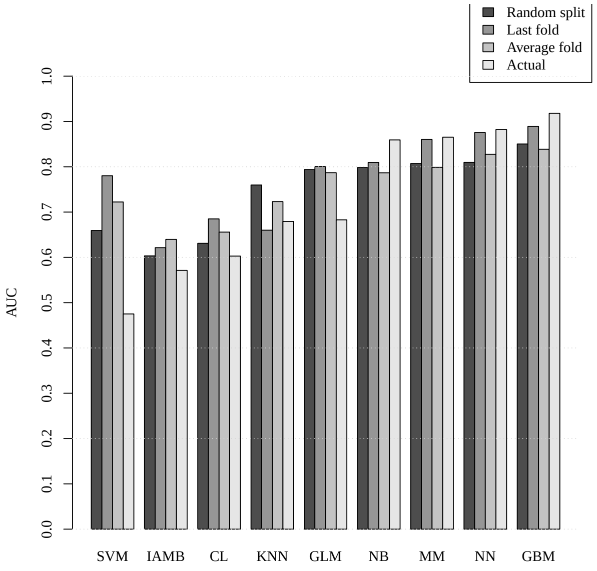

(a) Future 1-y predictions

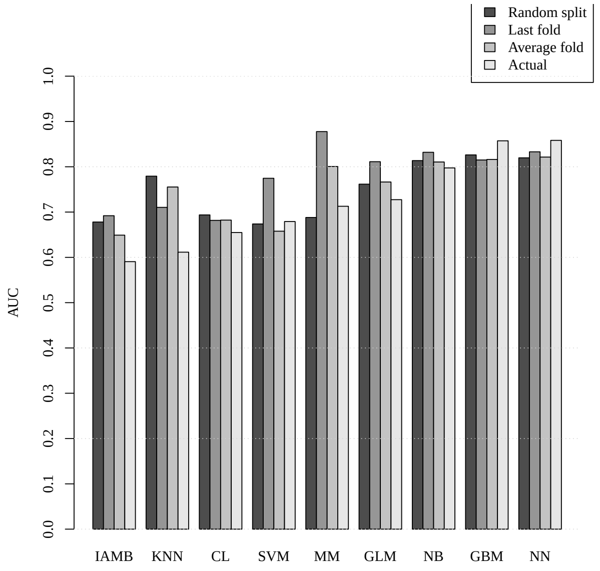

(b) Future 3-y predictions

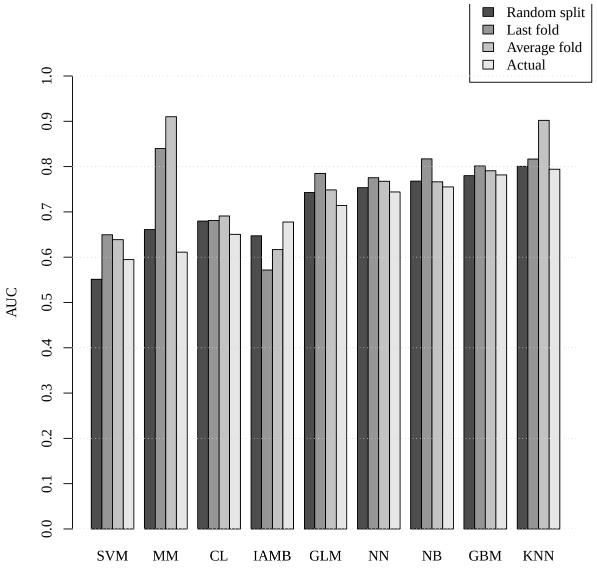

(c) Future 5-y predictions

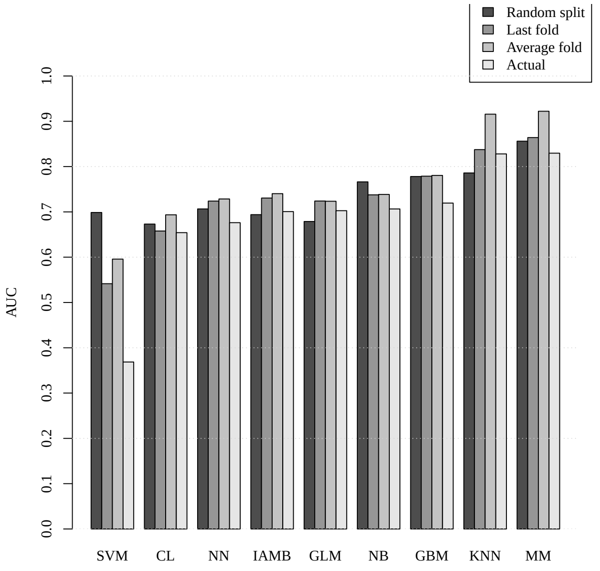

(d) Future 7-y predictions

Figure 10 – **AUC score on predicting infestations at years 2017 and 2018, and its estimations.** White, light gray, dark gray and black are the AUC scores on the test dataset (s), cross-validated AUC on the train dataset ($s^{\text{average-fold}}$), AUC on the last year of the train dataset ($s^{\text{last-fold}}$), and AUC on the test dataset obtained from a random partitioning of the training dataset into another train and test (s^{random}). The learners on are ordered from right to left on the x-axis based on their scores on the test dataset – *i.e.*, s (the white bars). **(a)**–**(d)** are future 1, 3, 5, and 7-y predictions.

Table 2 – Performance of the learners.

**length of future prediction** (r)	**Learners with** $s^{\text{average-fold}} \geq 0.8$	**Learner with the highest AUC on the test dataset** (s)	c^*	h^*	**AUC on the test dataset** (s)
1 year	GBM, NB, NN, MM	GBM	13	5	0.92
3 years	GBM, NN	NN	14	5	0.86
5 years	–	KNN	9	1	0.80
7 years	KNN, MM	MM	3	1	0.83

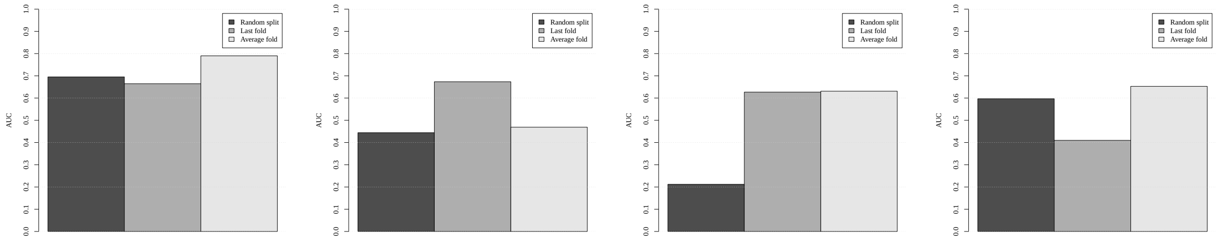

(a) $r = 1$ (b) $r = 3$ (c) $r = 5$ (d) $r = 7$

Figure 11 – **Accumulated absolute estimation error of the AUC score on years 2017 and 2018.** Light gray, dark gray and black are $\sum |s^{\text{average-fold}} - s|$, $\sum |s^{\text{last-fold}} - s|$, and $\sum |s^{\text{random}} - s|$. **(a)–(d)** are future 1, 3, 5, and 7-y predictions.

19 AUC results over different number of covariates and history lengths

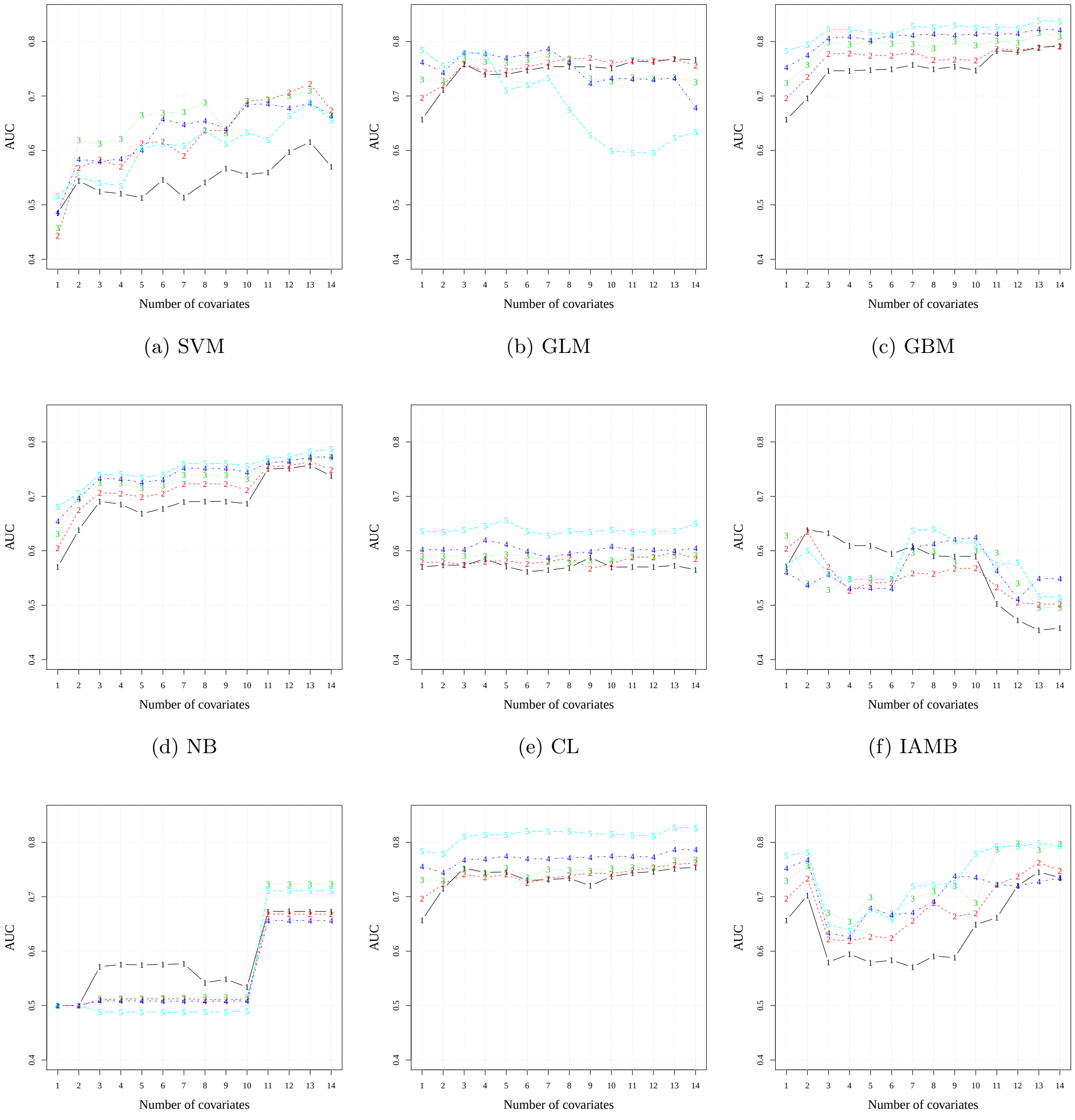

(a) SVM (b) GLM (c) GBM

(d) NB (e) CL (f) IAMB

(g) KNN (h) NN (i) MM

Figure 12 – **Future 1 year prediction.**

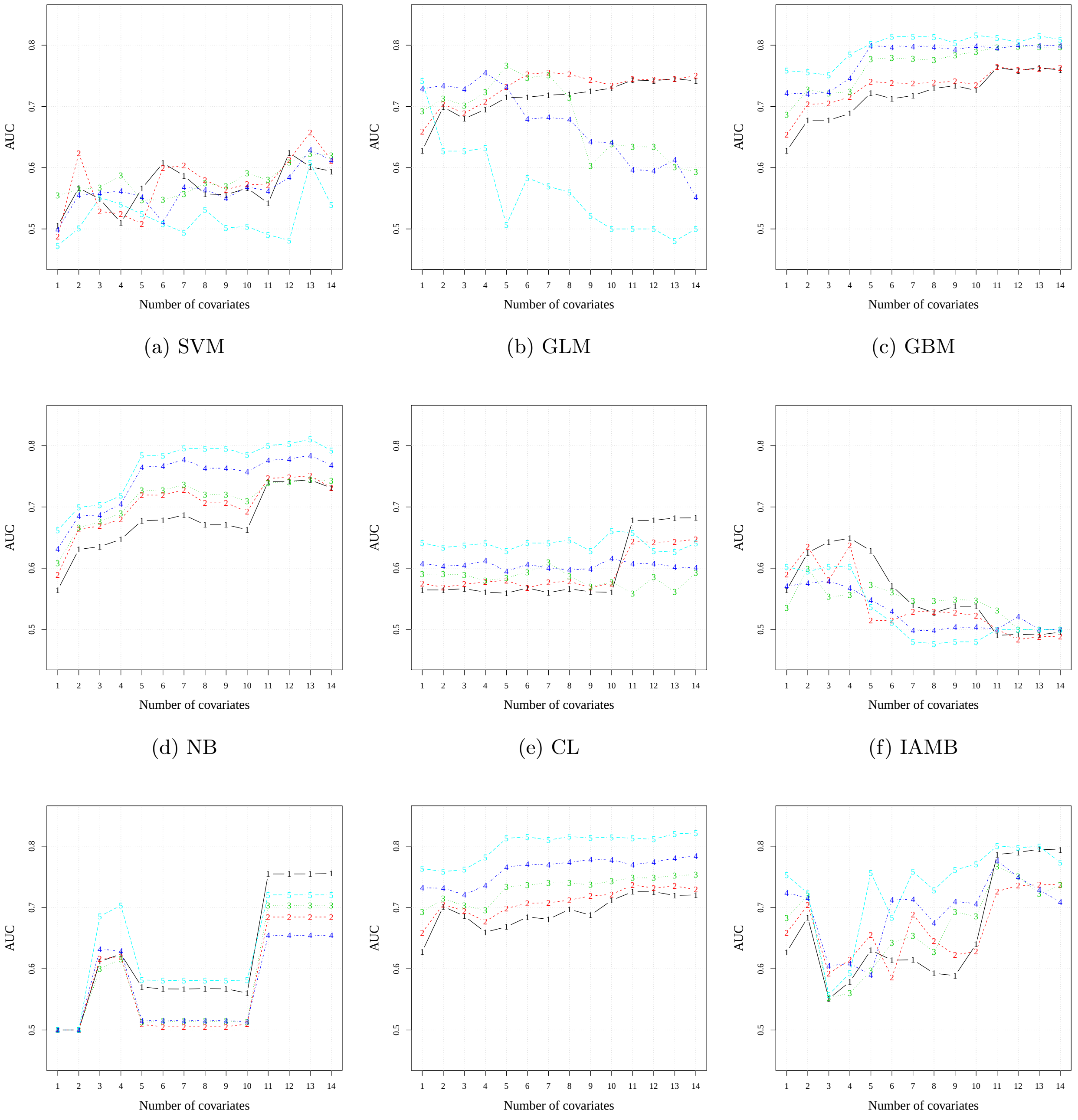

(a) SVM (b) GLM (c) GBM

(d) NB (e) CL (f) IAMB

(g) KNN (h) NN (i) MM

Figure 13 – **Future 3 years prediction.**

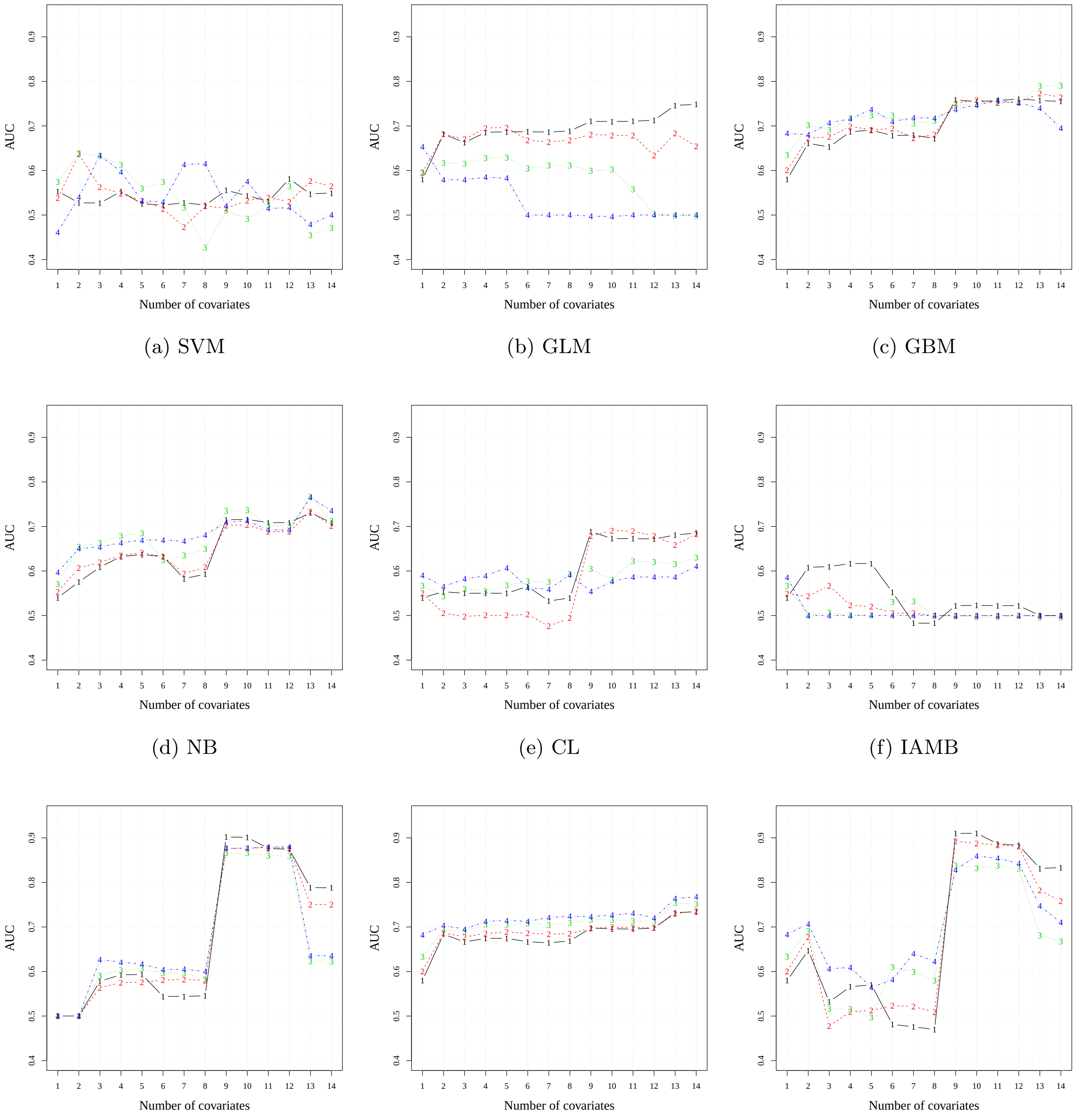

(a) SVM (b) GLM (c) GBM

(d) NB (e) CL (f) IAMB

(g) KNN (h) NN (i) MM

Figure 14 – **Future 5 years prediction.**

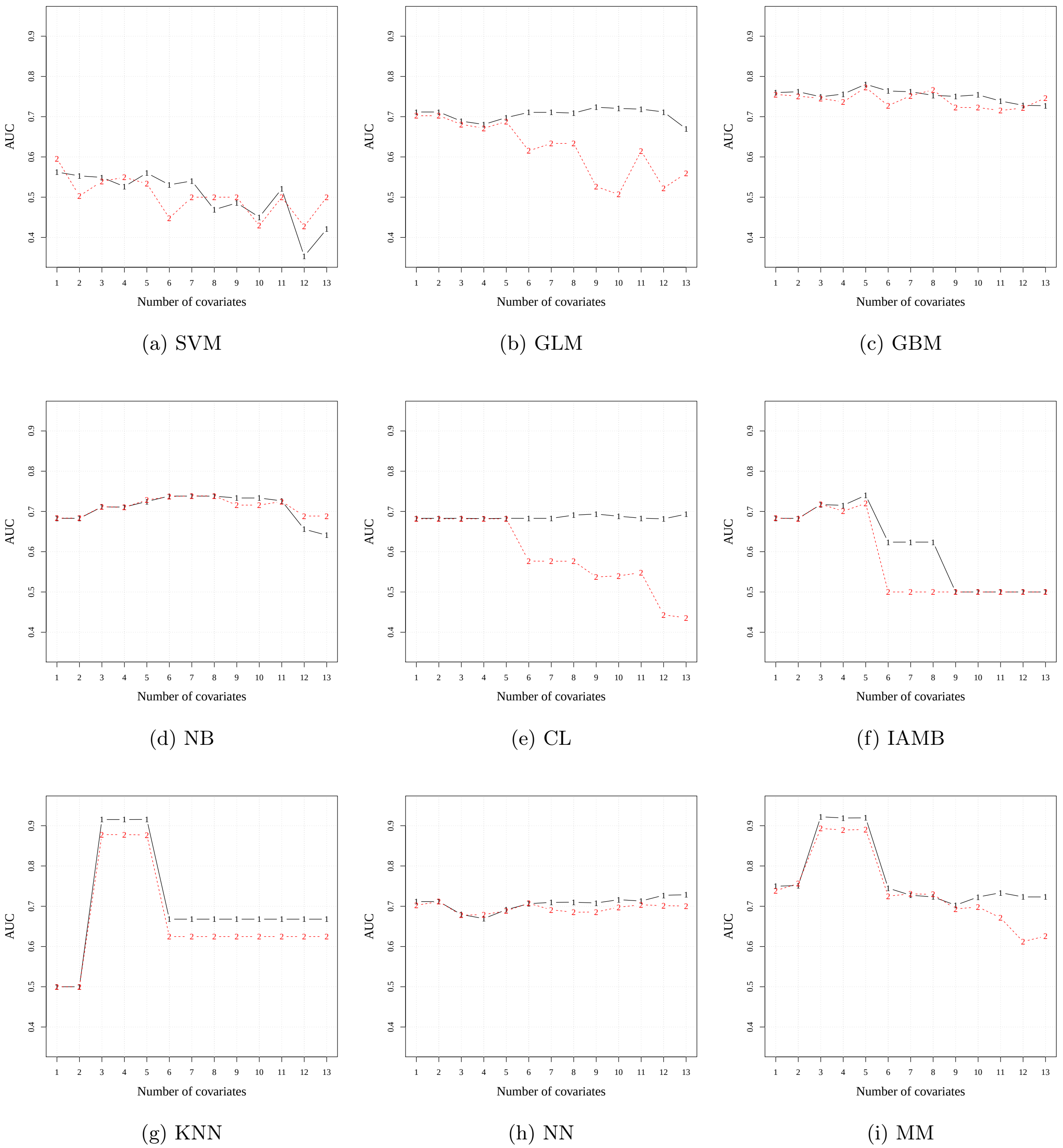

(a) SVM (b) GLM (c) GBM

(d) NB (e) CL (f) IAMB

(g) KNN (h) NN (i) MM

Figure 15 – **Future 7 years prediction.**

References

Chow, C. & Liu, C. (1968) Approximating discrete probability distributions with dependence trees. *IEEE transactions on Information Theory*, **14**, 462–467.

Haykin, S. (1994) *Neural networks: a comprehensive foundation.* Prentice Hall PTR.

Koller, D. & Friedman, N. (2009) *Probabilistic Graphical Models: Principles and Techniques.* MIT press.

Kunegel-Lion, M., McIntosh, R.L. & Lewis, M.A. (2019) Management assessment of mountain pine beetle infestation in Cypress Hills, SK. *Canadian Journal of Forest Research*, **49**, 154–163. doi: 10.1139/cjfr-2018-0301.

R Core Team (2018) *R: A Language and Environment for Statistical Computing.* R Foundation for Statistical Computing, Vienna, Austria.

Ramazi, P., Kunegel-Lion, M., Greiner, R. & Lewis, M.A. (under review) Modelling infestations using Bayesian networks. *Methods in Ecology and Evolution.*

Ridgeway, G. (2015) *gbm: Generalized Boosted Regression Models.*

Rong, X. (2014) *deepnet: deep learning toolkit in R.*

Rosiers, W. (2015) *parallelSVM: A Parallel-Voting Version of the Support-Vector-Machine Algorithm.*

Scutari, M. (2010) Learning Bayesian Networks with the bnlearn R Package. *Journal of Statistical Software*, **35**, 1–22. doi: 10.18637/jss.v035.i03.

Tsamardinos, I., Aliferis, C.F., Statnikov, A.R. & Statnikov, E. (2003) Algorithms for Large Scale Markov Blanket Discovery. *FLAIRS conference*, vol. 2, pp. 376–380.

Venables, W.N. & Ripley, B.D. (2002) *Modern Applied Statistics with S.* Springer, New York, 4th edn.
